# Atomic-scale engineering of ferroelectric-ferromagnetic interfaces of epitaxial perovskite films for functional properties

**DOI:** 10.1038/s41598-017-10194-4

**Published:** 2017-09-06

**Authors:** Simon Hausmann, Jingfan Ye, Toshihiro Aoki, Jian-Guo Zheng, Jochen Stahn, Francis Bern, Binda Chen, Carmine Autieri, Biplab Sanyal, Pablo D. Esquinazi, Peter Böni, Amitesh Paul

**Affiliations:** 1 Technische Universität München, Physik-Department, Lehrstuhl für Neutronenstreuung, James-Franck-Strasse 1, D-85748 Garching, Germany; 2 Division of Superconductivity and Magnetism, University of Leipzig, D-04103 Leipzig, Germany; 3 Laboratory for Neutron Scattering and Imaging, Paul Scherrer Institut, CH-5232 Villigen, Switzerland; 4 Department of Physics and Astronomy, Uppsala University, Box 516, SE-75120 Uppsala, Sweden; 5 Irvine Materials Research Institute, University of California-Irvine, Irvine, CA 92697-2800 USA

## Abstract

Besides epitaxial mismatch that can be accommodated by lattice distortions and/or octahedral rotations, ferroelectric-ferromagnetic interfaces are affected by symmetry mismatch and subsequent magnetic ordering. Here, we have investigated La_0.67_ Sr_0.33_ MnO_3_ (LSMO) samples with varying underlying unit cells (uc) of BaTiO_3_ (BTO) layer on (001) and (110) oriented substrates in order to elucidate the role of symmetry mismatch. Lattice mismatch for 3 uc of BTO and symmetry mismatch for 10 uc of BTO, both associated with local MnO_6_ octahedral distortions of the (001) LSMO within the first few uc, are revealed by scanning transmission electron microscopy. Interestingly, we find exchange bias along the in-plane [110]/[100] directions only for the (001) oriented samples. Polarized neutron reflectivity measurements confirm the existence of a layer with zero net moment only within (001) oriented samples. First principle density functional calculations show that even though the bulk ground state of LSMO is ferromagnetic, a large lattice constant together with an excess of La can stabilize an antiferromagnetic LaMnO_3_-type phase at the interface region and explain the experimentally observed exchange bias. Atomic scale tuning of MnO_6_ octahedra can thus be made possible via symmetry mismatch at heteroepitaxial interfaces. This aspect can act as a vital parameter for structure-driven control of physical properties.

## Introduction

In heterostructural interfaces of ABO_3_ perovskite-type structures, the misfit stress can be accommodated easily by strain induced via BO_6_ deformations through Jahn-Teller (J-T) distortions or cation displacement. Distinct BO_6_ rotational patterns of the individual layers, either in the substrates or the underlying layer will impose epitaxial strain^1^. Epitaxial mismatch can often be accommodated by bond stretching and octahedral distortions and/or rotations. For example, magnetic dead layers or antiferromagnetic (AFM﻿) layers are predicted at the SrTiO_3_ (STO)/La_0.67_ Sr_0.33_ MnO_3_(LSMO) interfaces owing to the strain induced distortion of the MnO_6_ octahedra^2,3^. However, besides epitaxial mismatch, the interface will experience the so-called symmetry mismatch. Effect of such mismatch on octahedral distortions is usually observed within a few unit cells (uc) from the interface. Symmetry mismatch alone can also affect subsequent magnetic ordering at the interface. Very recently, symmetry-mismatch of the oxygen octahedra at the CaRu*O*_3_/CaMn*O*_3_ interface was shown to switch off ferromagnetism^4^. Alteration of Mn-O-Ru bonding was proposed to reduce the orbital overlap and suppression of electron transfer stabilizes the AFM order in such systems.

The perovskite manganite LSMO is widely used in spintronics because of its half metallic behavior and Curie temperature T_*C*_ of around 370 K. Epitaxial strain, provided by an underlying material like BaTiO_3_ (BTO) has been often seen to influence T_*C*_. The strong magnetoelectric effect that exists in bilayers of LSMO-BTO can be due to the compressive strain in the BTO film that is induced when they are grown on STO substrates^5–7^. One may note that the existence of a non-magnetic interfacial layer is common only for LSMO grown on (001) substrates^8,9^ which is also more strained. As the underlying (001) BTO films get thinner, roughly below 10 uc the unit cell symmetry is lowered. This uc decrease may affect the octahedral deformation of the TiO_6_ octahedra^10^, followed by subsequent distortions or canting of the MnO_6_ octahedra. Additional octahedral distortions at the interface could originate from dissimilar MO_6_ rotational patterns or symmetry mismatch between the underlying BTO and the coherently grown LSMO layer due to J-T like distortions. For thicker (>10 uc) BTO layers, TiO_6_ distortions would be obviously absent. Hence the effect of lattice strains for (001) oriented LSMO samples can be reduced by growing them on top of a thicker BTO layer with relaxed strain, rather than growing them with (110) orientation. Subsequently, the magnetic properties are affected differently due to the different Mn-O-Mn bond angles and bond lengths at the interface^1^.

In this paper, we have investigated LSMO layers on top of BTO layers of different thickness, grown on (001) and (110) oriented STO substrates. For samples grown on (001) STO, grown systematically with increasing BTO uc, we do not observe significant lattice strains in the BTO layer around a certain thickness of 10 uc as seen by scanning transmission electron microscopy. Local lattice distortions of the (001) LSMO uc within the first few unit cells can be seen as an effect of the broken symmetry at the interface rather than lattice mismatch which is relevant for 3 uc of BTO. By measuring the magnetization along the [110]/[100] direction, we found exchange bias coupling at the LSMO-BTO interface. The exchange bias effect is absent in bilayers below a BTO thickness of around 10 uc, and also above. Polarized neutron reflectivity, with an applied magnetic field along the [110] direction, could identify a magnetic dead layer or an antiferromagnetic layer of around few nm thick between the heterointerfaces of BTO and LSMO. For samples grown on (110) STO substrates, no such non-magnetic layer is identified. First principles density functional calculations show that a large lattice constant (larger than in the bulk) stabilizes the LaMnO_3_-type antiferromagnetic phase at the interface with an excess of La. The ferromagnetic/antiferromagnetic (﻿FM/AFM) interface created is held responsible for the observed exchange bias along the [110]/[100] direction. Thus one can expect tailored functionalities which is not necessarily a consequence of strain engineering but guided by near neighbor exchange and symmetry mismatch.

## Results and Discussions

### Specimen design

In our investigation we have synthesized different samples with different thicknesses of BTO. To demonstrate how our comprehensive methodology works, we deposited on TiO_2_ terminated STO (001) and (110) substrates separately in two batches.

The first batch comprised of two samples, BTO n (001) (S1): [BTO_*n*_/LSMO_*m*_] on STO (001) and BTO n (110) (S2): [BTO_*n*_/LSMO_*m*_] on STO (110) where n = 20 and m = 27 are the numbers of uc The second batch comprised of five samples, BTO n (001) (S3–S7): [BTO_*n*_/LSMO_*m*_] on STO (001). Thus in the second batch the samples were composed of a (BTO)_*n*_/(LSMO)_*m*_ bilayer unit, with the BTO thickness systematically varying from 3–250 uc (n = 3, 6, 10, 25, 250), and the LSMO fixed at 39 uc (m = 39). Note that the thickness of the BTO films should be below 0.1 *μ*m to obtain high capacitance for the devices such as capacitors and ferroelectric random access memory. The thickness over which substantial strains can be maintained is limited to a few nanometers only. One may note that a full strain relaxation typically occurs above a thickness of 100 nm. Initial sample characterizations^11^ were done using X-ray reflectivity (XRR) and X-ray diffraction (XRD) and has been shown in the supplementary section.

### Scanning transmission electron microscopy

#### LSMO/BTO bilayer grown on (001) STO

**Figure 1:**
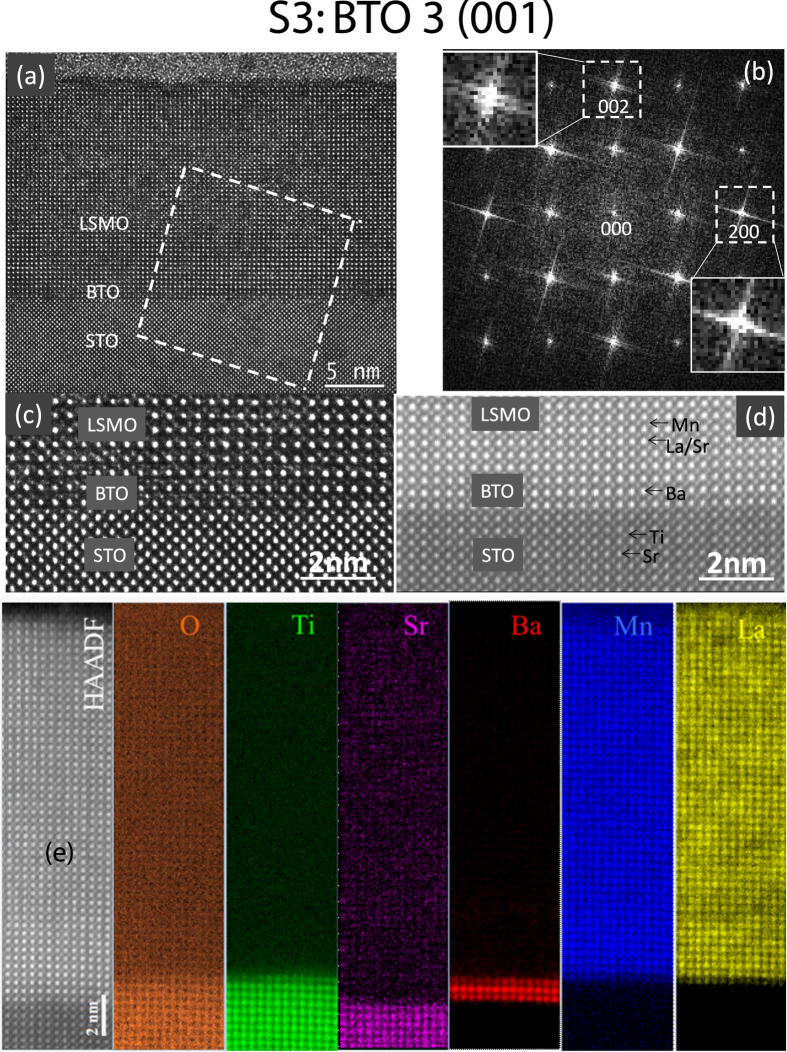
Atomic structure of specimen S3. (**a**) HRTEM image of cross-sectional specimen S3, showing LSMO and BTO layers on the STO substrate. (**b**) FFT pattern of the area marked by the dashed square in (**a**) containing BTO, LSMO and STO. (**c**) Enlarged HRTEM image at the interfaces. (**d**) Z-contrast image of the interfaces. (**e**) Atomic resolution HAADF STEM image and EELS maps of O, Ti, Sr, Ba, Mn and La.

Cross-sectional TEM experiments were conducted on three samples (S2, S3 and S5) to examine the microstructures of LSMO/BTO films grown on STO substrates with different orientations. First we focus on the samples S3 and S5. Figure 1(a) shows the HRTEM lattice image of sample S3, where BTO film with the thickness of 3 uc was grown on the (001) STO substrate followed by LSMO film. The interface between STO and BTO is atomically abrupt and can be readily identified, while BTO and LSMO films do not show clear interface contrast. The image is featured with dots forming approximate squares in all areas from the substrate to the film, indicating the characteristics of [010] zone axis image and high quality epitaxial film growth. This zone axis is further confirmed by Fast Fourier Transformation (FFT) pattern of the lattice image. Figure 1(b) is the FFT pattern of the area containing BTO, LSMO and STO, as marked in the dashed square in Fig. 1(a). It is obvious that the 002 reflections of three materials are split, while 200 reflections are almost superimposed, implying that there must be in-plane strain in the films. Figure 1(c) is an enlargement of the interfaces. It is clearly seen that the lattice planes continue across the interface indicating that films were grown epitaxially on the STO substrate.

The in-plane lattice parameters of bulk BTO and STO are *a* = 3.994 Å and 3.905 Å, respectively, the misfit between BTO and STO is 2.28%, that is, if the misfit was relaxed fully by misfit dislocations, then there would be a misfit dislocation within 44 unit cells. The misfit between BTO and LSMO is even larger (about 3.20%), misfit dislocations should occur in a shorter distance. However, in Fig. 1(a) which displays a width of more than 80 unit cells, no misfit dislocations were observed. Misfit dislocations were hardly observed at the interfaces between the neighboring materials. Therefore, the misfit between different materials is accommodated primarily by strain. Figure 1(d) is a high angle annual dark field (HAADF) STEM image of the interface area. Besides the coherent interface feature, the image confirms that the BTO film was grown on the Ti-O terminated STO (001) surface, as designed.

A combination of atomic resolution HAADF STEM and electron energy loss spectroscopy (EELS) maps of O, Ti, Sr, Ba, Mn and La, shown in Fig. 1(e), was used to reveal atomic arrangement across the interfaces directly. Three layers of Ba atoms in the Ba map confirm that BTO film thickness is of 3 uc It is noted that the middle layer of Ba is brighter than other two layers, indicating the intermixing of Ba and La in the upper layer and intermixing of Ba and Sr in the lower layer. Despite the small intermixing, both interfaces are very sharp.

**Figure 2:**
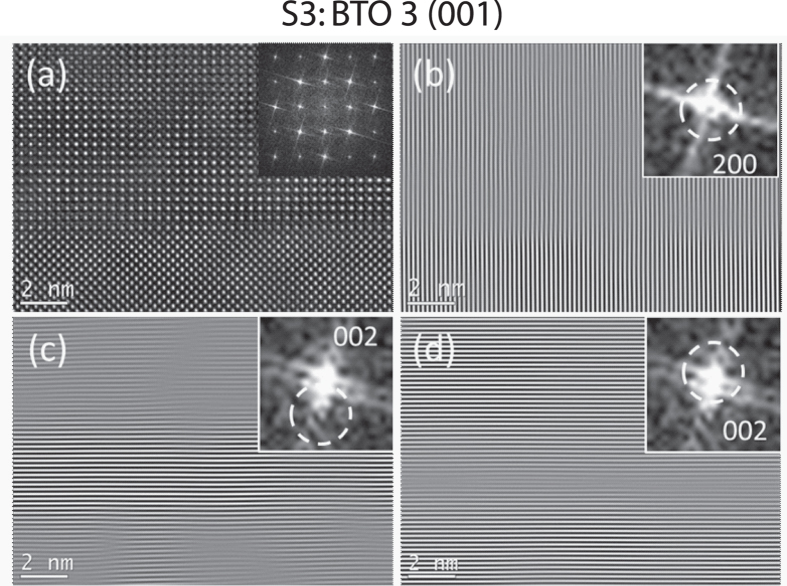
Atomic structure of specimen S3. (**a**) Enlarged HRTEM image of S3 and its FFT pattern (inset). (**b**) Reconstructed image using the dashed circles around ±200 reflections and showing 200 fringes in the image and 200 reflection in FFT inset. (**c**) Reconstructed image using the dashed circles around ±002 reflections of BTO. The bright band contains BTO 002 fringes and 002 reflection is shown in the inset. (**d**) Reconstructed image using the dashed circles around ±002 reflections of LSMO and BTO. The two bright bands contain 002 fringes of LSMO and BTO, respectively, and 002 reflection is shown in the inset.

As we know, BTO has lattice parameters larger than STO and LSMO. The introduction of a thin BTO film between STO and LSMO should result in the in-plane tensile strain in both STO and LSMO and in-plane compressive strain in BTO. These opposite strains favors the similar in-plane lattice parameters of these materials. This is why the 200 reflections of the three materials are almost superimposed (Fig. 1(b)). Figure 2(a) shows the enlarged HRTEM image and the corresponding FFT pattern of S3. In the reconstructed image (Fig. 2(b)) using the pairs of ±200 reflections, one of which is marked in the dashed circle of the inset, the (200) lattice fringes continue across the interfaces, which further confirms the nature of coherent interface. To keep the uc volume constant, the in-plane strains should be correlated with the change of out-of-plane lattice parameters, that is, the out-of-plane lattice parameter of LSMO should become smaller while the corresponding parameter of BTO becomes bigger. The out-of-plane lattice parameters are associated with the split 002 reflections showed in Fig. 1(b). We reconstructed images using different 002 reflections showed in the dashed circles in the insets of Fig. 2(c,d), respectively. Figure 2(c) displayed the bright band containing BTO with larger lattice parameter and Fig. 2(d) highlights two bright bands containing STO and LSMO with smaller out-of-plane lattice parameters. It should be noted that the boundary between bright band and the weaker band is not the chemical boundary, but the boundary between areas with slightly different interplanar spacings.

**Figure 3:**
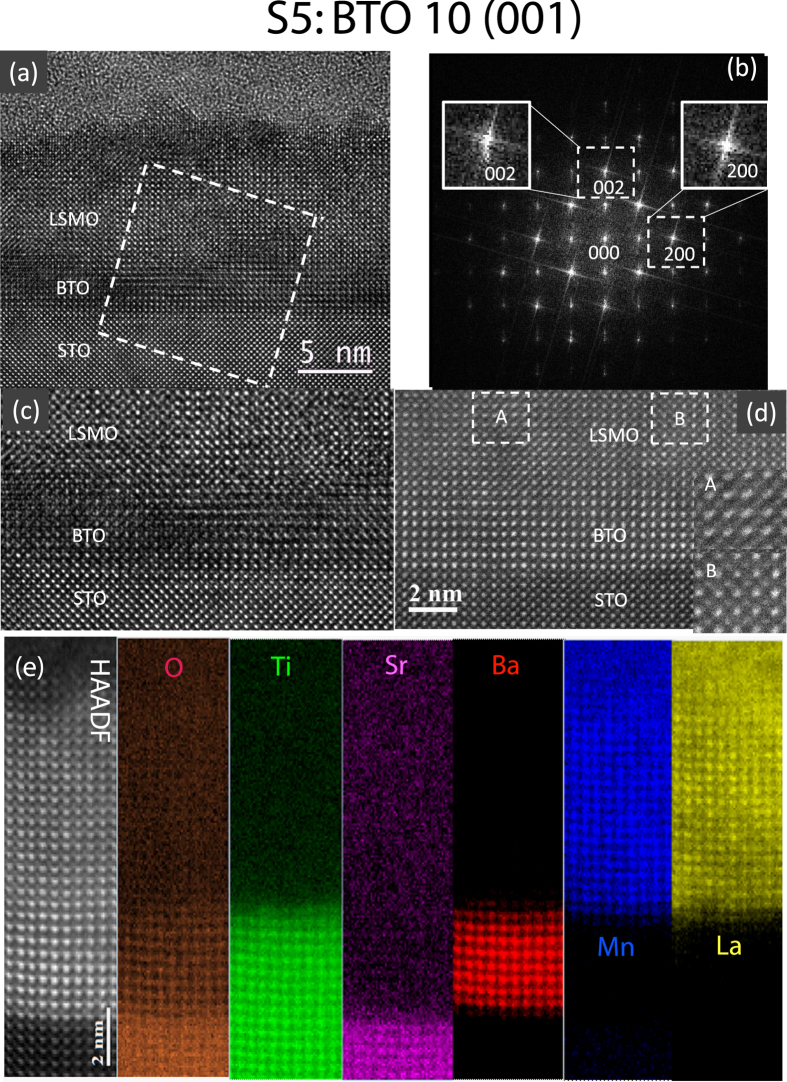
Atomic structure of specimen S5. (**a**) HRTEM image of cross-sectional specimen S5, showing LSMO and BTO layers on the STO substrate. (**b**) FFT pattern of the area marked by the dashed square in (**a**) containing BTO, LSMO and STO. (**c**) Enlarged HRTEM image at the interfaces. (**d**) Z-contrast image close to the interfaces. The difference between nearby LSMO areas such as A and B (insets) are clearly shown, indicating local lattice distortion in A-area. (**e**) Atomic resolution HAADF STEM image and EELS maps of O, Ti, Sr, Ba, Mn and La.

Similar TEM experiments were done on sample S5 with slightly thicker BTO film between the STO (001) substrate and LSMO film. The sample S5 has some similarities to sample S3. Figure 3(a) is a cross-sectional HRTEM lattice image of sample S5 observed along the [010] zone axis, as is confirmed by its FFT pattern (Fig. 3(b)). The in-plane lattice parameters of the three materials are close to each other, while the out-of-plane lattice parameters show visible difference. The interface between STO and BTO is atomically abrupt (Fig. 3(a,c,d)), and the Z-contract image (Fig. 3(d)) shows much clear atomic arrangement at the interface. These images indicate that both BTO and LSMO films are epitaxially grown and the interfaces are coherent. Besides BTO film thickness, Sample S5 has other different features from sample S3, for example, the local lattice distortions in the LSMO film. The difference between nearby LSMO areas such as A and B marked in Fig. 3(d) is obvious. Their enlargements are displayed in insets A and B, respectively. Inset B shows a normal [010] Z-contrast image of LSMO where larger bright spots correspond to La/Sr positions and smaller bright spots to Mn positions, while the diagonal elongation of the spots in inset A is readily seen. Areas A and B are located nearby, so the difference should not be caused by imaging conditions, but the local lattice distortion of uc in the beam direction.

The atomic arrangement at the interfaces in sample S5 is displayed in Fig. 3(e). Because elemental mapping takes much longer time than normal Z-contract STEM image, the sample drift can cause the image of atomic planes slight bending, but it is easy to identify atomic layers. There are about 10 Ba atomic layers between STO and LSMO. Intermixing of Sr/Ba and Ba/La at the interfaces occur within a range of a couple of uc or about 1 nm.

**Figure 4:**
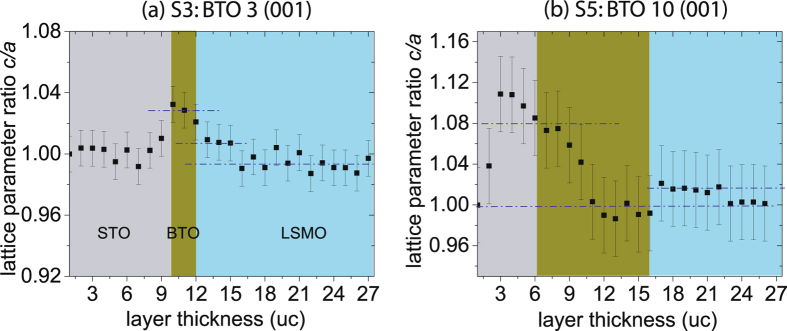
Lattice parameter ratio of specimens S3 and S5. The plot of the extracted ratio *c*/*a* of the lattice parameters as a function of the number of uc from the Z-contrast images of cross-sectional TEM specimens of (**a**) S3 and (**b**) S5. Each data point was obtained by averaging a set of 10 measurements from unit cells arranged parallel to the interface. The error bars indicate the standard error of the measurements. The blue dashed lines are a guide to the eye.

It has been shown earlier that lack of rotations at the LSMO/STO (001) interface and subsequent unusual *c*-axis lattice expansion (by 0.025 Å) is not caused by conventional lattice mismatch but is rather induced by crystallographic symmetry mismatch between LSMO and STO structures across the interface^12^. In ultrathin LSMO films grown on (110)-oriented STO substrates, however, the octahedral coupling across the interface induces distinctive BO_6_ octahedral distortions leading to lattice mismatch rather than symmetry mismatch. We have measured the in-plane lattice parameter along the *a*-axis and out-of-plane lattice parameter along the *c*-axis as a function of layer uc from the Z-contrast images of cross-sectional TEM specimens of S3 and S5 (see the supplementary section). For S3, the *c*/*a* ratio which is plotted in Fig. 4(a) shows a behavior typical of the consequences of lattice mismatch. It goes down from 1.03 at the STO-BTO interface to 1.0 within the LSMO layer near the BTO-LSMO interface and then stabilizes at 0.99, farther away. For S5, we find a *c*/*a* ratio, plotted in Fig. 4(b), which varies within the BTO layer from 1.08 at the STO-BTO interface to close to 1.0 near the BTO-LSMO interface. Within the LSMO layer, it increases to 1.01 at the interface before stabilizing at 1.0, farther away. The average *c*-axis parameter at the interface shows a jump from 3.892 Å within the BTO layer to 3.973 Å within the LSMO layer and is extended over for at least 6 uc away from the interface. This *c*-axis parameter expansion (by 0.081 Å) followed by reduction can be looked upon a direct consequence of symmetry mismatch at the BTO-LSMO interface and octahedral tilts farther away from the interface, respectively^12^.

#### LSMO/BTO bilayer grown on (110) STO

**Figure 5:**
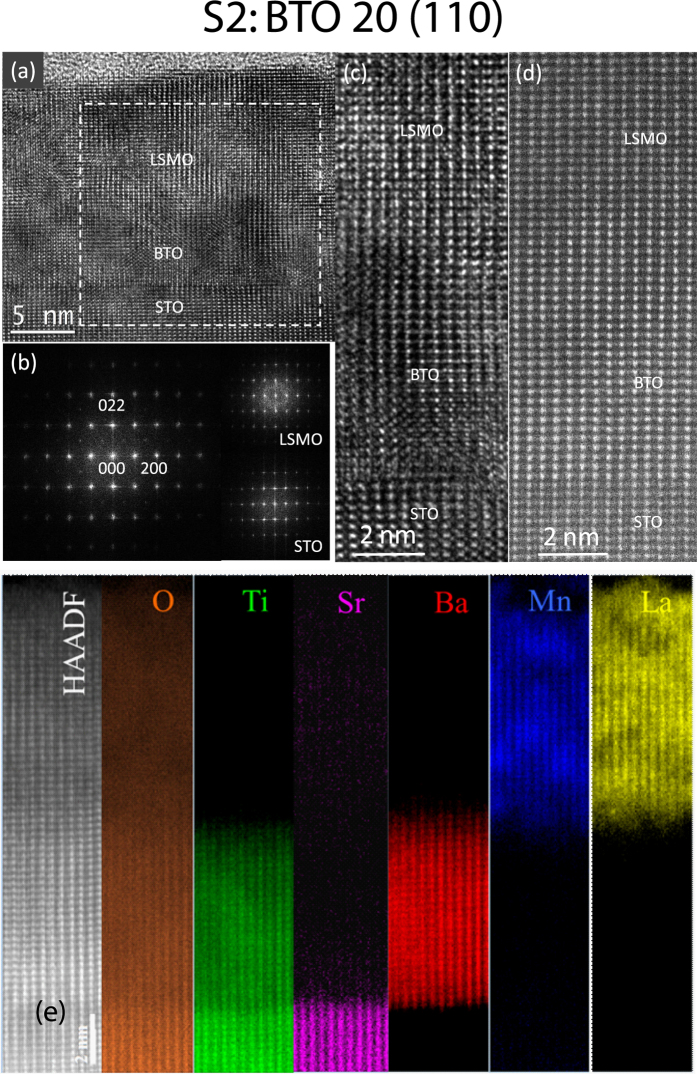
Atomic structure of specimen S2. (**a**) HRTEM image of cross-sectional specimen S2, showing LSMO and BTO layers on the STO substrate. (**b**) FFT pattern of the area marked by dashed square containing BTO, LSMO and STO. (**c**) Enlarged HRTEM image. (**d**) Z-contrast image. (**e**) Atomic resolution HAADF STEM image and EELS maps of O, Ti, Sr, Ba, Mn and La.

To understand the effect of the substrate on film microstructure, cross-sectional TEM experiments were carried out on LSMO/BTO films grown on (110) STO substrate as well. Figure 5(a) is the HRTEM lattice images of sample S2 taken when the electron beam is along the STO [0$\overline{1}$1] direction. The BTO film has an epitaxial growth on the STO substrate and the interface between STO and BTO is atomically abrupt. LSMO is also grown epitaxially on the BTO although their interface is not easily identified. The epitaxial growth can be seen from the FFT pattern (Fig. 5(b)) of the dashed square area in Fig. 5(a) containing the three materials. It appears that one set of diffractions spots in the [0$\overline{1}$1] zone axis except for the split of high index spots such as 033. This is because the FFT patterns from the STO, BTO and LSMO are quite similar. The inset at lower right corner is the FFT pattern of STO and the inset at upper right corner is related to LSMO. The FFT pattern of BTO (not shown) is almost the same as that of STO except very small split of their corresponding spots in the vertical direction, as shown in Fig. 5(b). So the major spots of three materials are almost overlapped. It should be mentioned that the weak extra reflections in Fig. 5(b) are related to LSMO, as confirmed in the inset at upper right corner. Figure 5(c) is the enlargement of HRTEM lattice image of all three materials. The phase contrast image is affected by imaging condition, such as sample thickness and local strain, but it is clearly seen that the lattice planes continue from the substrate to BTO and LSMO films. The Z-contrast image (Fig. 5(d)) shows the arrangement of heavy atoms much more clearly than the phase contrast image. The epitaxial growth of the films is evident. The elemental maps (Fig. 5(e)) show the BTO film thickness is about 8 nm. The interface between STO and BTO remains sharp (about one uc in thickness), while the interface between BTO and LSMO is diffused (about 2–3 nm in thickness), suggesting intermixing between BTO and LSMO at their interface. The sample S2 has a BTO/LSMO interface much thicker than those in samples S3 and S5. This can be related to either the substrate orientation on the BTO/LSMO interface or growth conditions. The La and Mn maps reveal some inhomogeneity. Some weaker La intensity areas correspond to the stronger Mn intensity areas, indicating the small variation of La/Mn ratio in the LSMO film.

### Magnetization

The magnetization measurements were done along two crystallographic axes: at 0° along [110] or along [001], both being along the y-axis for different substrates ﻿STO﻿﻿(001) and STO (1﻿10)and at 45° along [100] with respect to the y-axis, realized by rotating the magnetic field direction in the film plane of STO (001). An in-plane rotation showed an easy axis along the [110] direction^13^. The growth direction is designated along the z-axis.

**Figure 6:**
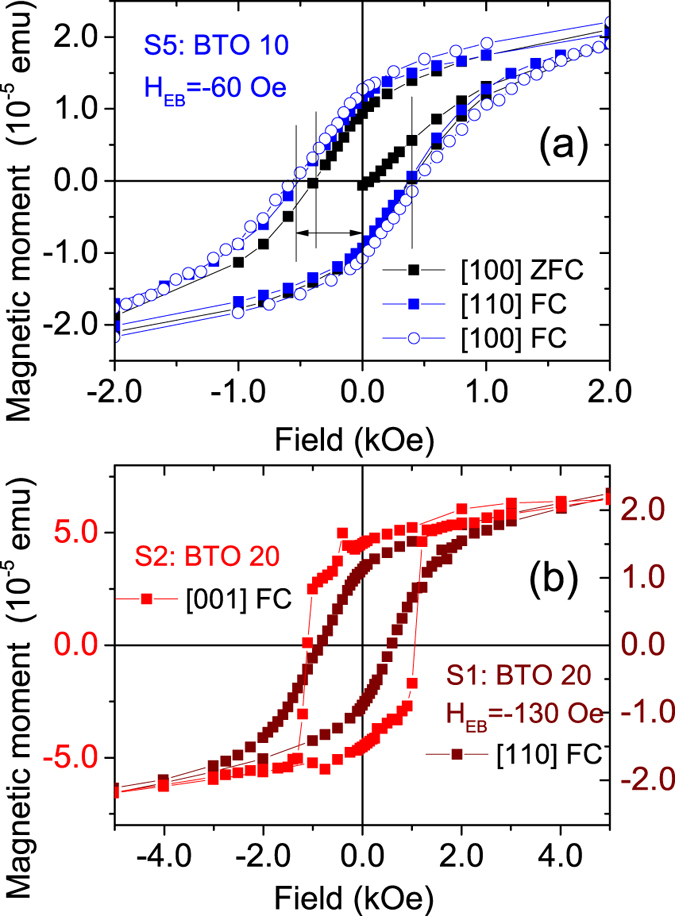
Magnetization measurements of S5, S1 and S2. (**a**) Hysteresis loop measurements at 10 K after field cooling (FC = 10 kOe) and measured along the [100] and [110] directions for the sample S5. The ZFC curve measured along the [100] direction is also shown for comparison. (**b**) Hysteresis loop measurements at 10 K for the sample S1 after field cooling and measured along the [100] and [110] directions and S2 after field cooling and measured along the [001] direction.

The magnetization data from the sample series can be seen in the supplementary section. The most significant feature is the loop shift along the magnetic field axis towards negative fields for S5 (${H}_{{\rm{EB}}}={H}_{{\rm{c}}}^{+\alpha }+{H}_{{\rm{c}}}^{-\alpha }$)/2 where ${H}_{{\rm{c}}}^{+\alpha /-\alpha }$ are the coercive fields for increasing and decreasing magnetic fields) with an exchange bias field *H*_EB_ ≈ −60 ± 3 Oe. Note that the saturation magnetization is lowest and the coercivity is highest for the sample S5 and the magnetic moment is calculated as 0.3 *μ*_*B*_/Mn atom. Also note that a similar exchange bias was observed when measured along the [100] axis as shown in Fig. 6(a) where we compare the two measurements at T = 10 K with respect to a zero field cooled curve. It is well known that the ferroelectric domain walls are orderly aligned along the BTO [110] direction suggesting the presence of the spontaneous ferroelectric polarization and subsequent magnetoelectric (﻿﻿ME) coupling between the ferroelectricity of BTO and the magnetism in LSMO^14^. Mn L-edge EELS profiles as a function of monolayers across the LSMO layers for S5 exhibit no significant shift in the energy or shape of the Mn L-edge (see the supplementary section) which indicates that the magnetic properties arise from structural modulations and not from charge-based effects.

Interestingly, there is no such exchange bias for S3, S4 and S7. The magnetization is slightly higher in S6 than in S5 which can be the cause of a smaller *H*_EB_(≈−7 ± 3 Oe) in S6. The disappearance of exchange bias in samples with lower BTO thickness, lower than that in S5, indicates an effect of interface modification due to strain. It has been suggested earlier that difference in octahedral rotation symmetry at the heterostructural interface causes discontinuity of the rotation leading to new phases which can affect the magnetism^15^. For samples with higher BTO thickness is due to increased interdiffusion (following the XRR data). The effect of interdiffusion can be seen from the EELS elemental profiles of S3 and S2 (shown in the supplementary section). For S3 (3 uc), the interface is sharp and the interdiffusion is less than 1 nm. The interdiffused layer in S2 (20 uc) is around 5 nm. Thus, with an increase in BTO thickness the possibility of interdiffusion becomes higher and this interface modification can eventually destroy the exchange coupling.

Though the two 001 oriented samples S1 (20 uc) and S6 (25 uc) are apparently similar, the exchange bias for S6 is comparatively smaller than that for S1. The thickness of LSMO is also different for the two. Increased BTO uc can induce a higher interdiffusion at the BTO/LSMO interface in S6 as compared to that in S1. This is indeed the case as S1 has a thinner interdiffused layer than S2 and S6 (following the XRR data in the supplementary section). Interdiffusion causes interface modification to destroy the exchange coupling. Moreover, the symmetry mismatch or lattice distortion can be drastically different due to the presence of additional five uc of BTO across the 001 heterointerface. Mn valence is influenced by several factors such as local composition, strain, magnetic structure and phase segregation. Local Sr/La ratio change is a major reason to affect Mn valence. Though the oxygen concentration does not display any significant change from BTO to LSMO for one batch of samples (S1, S2 and S5) from another (S3, S4, S6 and S7), local La inhomogeneity within S2 was clearly evident which may affect the magnetism drastically (see the Mn L_3_/L_2_ white line intensity ratio from the EELS profiles that were used to characterize Mn valence with respect to sample S2 in the supplementary section, as an example). In spite of different orientation, we expect similar local inhomogeneity in S1 as it is from the same batch and with fairly similar BTO thickness. Thus, S1 and S6 can have different magnetic couplings.

Another scenario is observed in Fig. 6(b) for the samples S1 and S2 of the first batch where the saturation fields are lower. For example, the sample S1 has an exchange bias field of around–130 ±3 Oe when measured along the [110] direction. The sample S2, however, did not show any exchange bias when measured along the [001] direction. Thus, even though there exists a difference in interdiffusion, the disappearance of exchange bias here has a predominant orientational dependence.

### Polarized neutron reflectivity (PNR)

**Figure 7:**
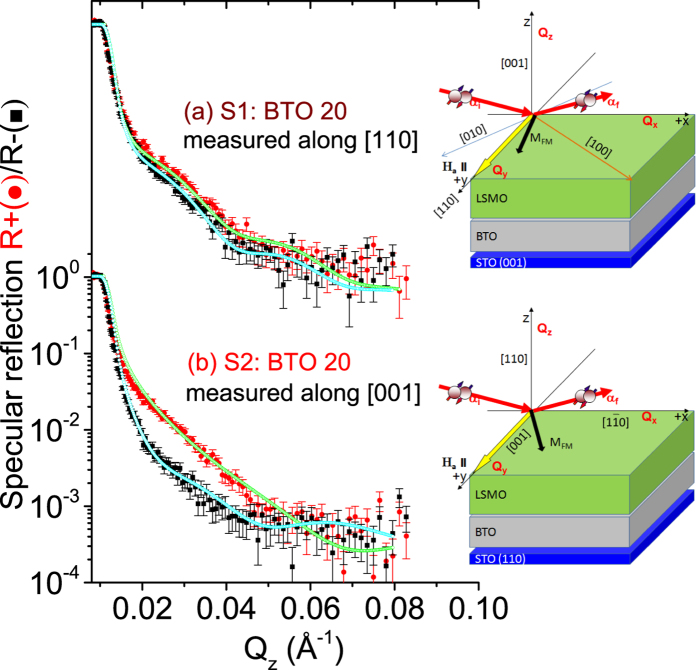
PNR measurements of specimens S1 and S2. Specular neutron reflectivity patterns (solid symbols) along with their best fits (open symbols) as a function of *Q*_z_ for the NSF [R_−_ (black) and R_+_ (red)] channels measured at a saturation field H_a_ = +5.0 kOe at 15 K for the sample (**a**) S1 along the [110] direction and the sample (**b**) S2 along the [001] direction. The measurements were performed using the Selene setup. Schematic of the magnetic field measurement and neutron scattering geometry along the [100] and [001] directions are shown alongside.

The effect of sample growth on different orientations of STO has also been investigated. Figure 7 shows the polarized neutron intensity profiles along *Q*_z_ and their fits for S1 (grown on STO (001)) and S2 (grown on STO (110)) after cooling the sample in a saturating field of +5.0 kOe and measuring at +5.0 kOe and at 15 K. The measurements were done using the Selene setup^16^.

The fits are done using a simple model of block-potentials. The parameters that were used for fitting are the individual layer thicknesses, the nuclear and magnetic SLDs of the individual layers. The errors in the thickness of the layers are ±0.2 nm, while that for the nuclear and magnetic scattering length densities *ρ*_*n*_ and *ρ*_*m*_ values are ±0.1 × 10^−6^ Å^−2^ and ±0.05 × 10^−6^ Å^−2^, respectively. The interface roughness is ≃0.5 ± 0.5 nm. The magnetic scattering length density of the top layer was fitted independently from the stack which was further divided into two more layers of LSMO. As the top layer is slightly Sr segregated^17^, we assumed a reduced nuclear scattering length density when compared with the stack. Fitted parameters were obtained using the minimization of chi squared (*χ* ^2^ or the goodness of fit) value.

For S1, the PNR data measured along the [110] direction show a small splitting of the R_+_ and R_−_ profiles, a signature of net magnetization within the sample. The magnetic moment is drastically reduced to 0.38 ± 0.05 *μ*_*B*_ per Mn atom (*ρ*_*m*_ = 0.17 × 10^−6^ Å^−2^) as compared to bulk LSMO and is plausibly accompanied by a non-magnetic interfacial layer. However, the overall low magnetic moment makes it difficult for a proper thickness estimate of the same. The sample S2 (grown on a (110) STO substrate), on the other hand, show a relatively large splitting of the R_+_ and R_−_ profiles when measured along the [001] direction. This indicates significant increase in magnetization to 1.79 ± 0.05 *μ*B per Mn atom (*ρ*_*m*_ = 0.8 × 10^−6^ Å^−2^) with a lower magnetization at the interface (0.4 ± 0.05 *μ*B per Mn atom), which nevertheless is still significantly low as compared to that is expected in bulk. Such low magnetic moments are often expected particularly for LSMO-BTO samples with similar numbers of uc^18^. The magnetic moments from the PNR data are always similar to that obtained from the SQUID data. In this sample, though the existence of a surface layer (4.5 ± 0.5 nm) with lower magnetization (0.22 ± 0.05 *μ*B per Mn atom) could be identified, no non-magnetic layer could be ascertained at the BTO/LSMO interface.

**Figure 8:**
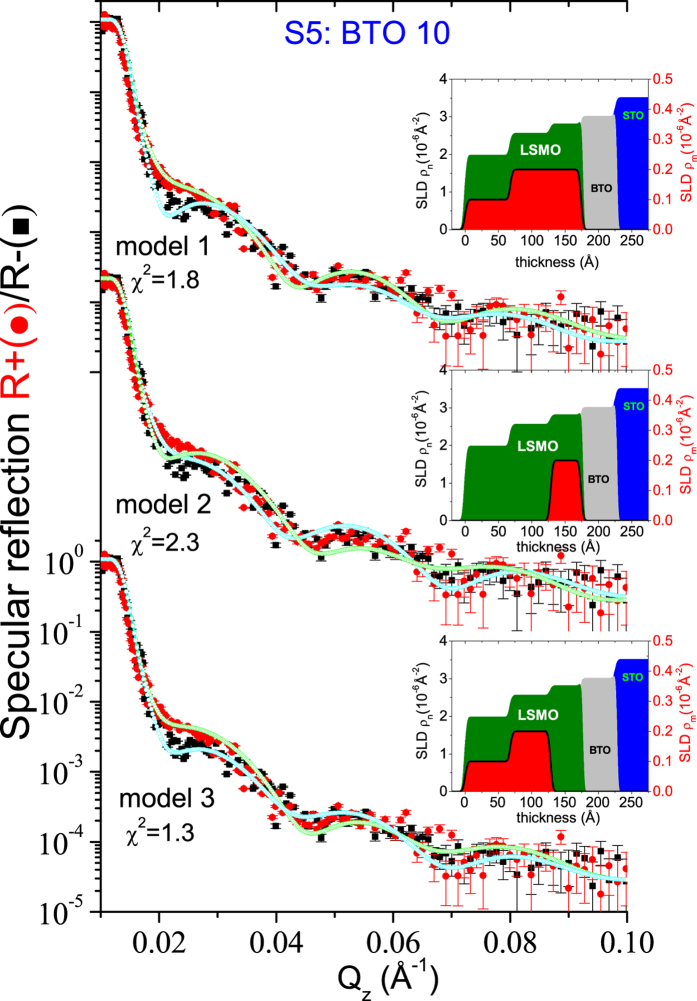
Model fittings for PNR data of specimen S5. Specular neutron reflectivity patterns (solid symbols) along with their best fits (open symbols) using three different models (1, 2, and 3) as a function of *Q*_z_ for the NSF [R_−_ (black) and R_+_ (red)] channels measured at a saturation field H_a_ = +5.0 kOe at 10 K for the sample S5 along the [110] direction. The nuclear (*ρ*_n_) and magnetic (*ρ*_m_) SLDs versus the thickness of the multilayer are also shown alongside.

In order to explore and confirm the existence of the non-magnetic layer at the interface, we choose sample S5 which showed maximum *H*_EB_ for PNR measurements without using the Selene set up. Figure 8 shows the polarized neutron intensity profiles along *Q*_z_ of S5 after cooling the sample in a saturating field of −5 kOe and measuring at +5.0 kOe and at 10 K. The room temperature data did not show any significant magnetic moment in the sample. The low temperature data did show a small splitting of the R_+_ and R_−_ profiles, a signature of net magnetization within the sample.

The magnetic moment of LSMO in S5 is divided into three regions within one layer. It is around 0.27 ± 0.05 *μ*_*B*_ per Mn atom (*ρ*_*m*_ = 0.12 × 10^−6^ Å^−2^) for the first 6.0 ± 0.5 nm and 0.56 ± 0.05 *μ*_*B*_ per Mn atom (*ρ*_*m*_ = 0.25 × 10^−6^ Å^−2^) for the next 6.5 ± 0.5 nm and the rest of the layer (≈4.5 ± 0.5 nm) is non-magnetic. The strongly reduced magnetic moment as compared to the maximum value for a purely ferromagnetic ordering of LSMO, as obtained from the PNR data, indicates a strong tendency towards an antiferromagnetic order. To show the validity of our fits we have considered three different possible models (model 1, model 2 and model 3) and show them alongside in Fig. 8. The obtained average SLD profiles that have been extracted from the PNR fits. The model 1 assumes that the magnetization is throughout the entire thickness of the LSMO layer. The model 2 assumes a magnetization only at the interface of LSMO-BTO and zero for the rest of the LSMO layer. Model 3 assumes an intermediate layer between BTO and LSMO that is completely antiferromagnetic (no net magnetic moment). The thickness of the non-magnetic layer is around 4.5 ± 0.5 nm. The best fit is obtained using the model 3. Note that the reflectivity drops by at least five orders of magnitude over a relatively large *Q*_z_ range of 0.12 Å^−1^ due to the high signal to noise ratio at AMOR, as compared to another PNR data on similar systems, that was reported by Alberca *et al*.^13^, where it drops by three orders of magnitude. The data quality has enabled us to figure out the variation in the nuclear SLD within the LSMO layer and particularly the existence of a magnetic dead layer in the system. It may be noted that a magnetic moment of 1.11 ± 0.05 *μ*_*B*_ per Mn atom was found for the S3 sample from the PNR data at 10 K (not shown). Thus, the BTO layer thickness is seen to be strongly influencing the magnetization of the LSMO layer on top.

#### Magnetization in (001) and (110) oriented LSMO

Unlike ordinary itinerant ferromagnets, the spin manganites are sensitive to the Mn-O-Mn bond and the local density of orbital states as both have orientational dependencies^6^. For in-plane (001) LSMO-BTO interface, while every MnO_6_ octahedra has one TiO_6_ octahedra as its neighbor, for (110) interface, every MnO_6_ octahedra has two TiO_6_ octahedra. The larger the number of neighboring TiO_6_ octahedra, higher is the expectation of localized electrons and stronger is the Mn-O-Mn double-exchange (DE) coupling, resulting in stronger magnetic moments. For (110) oriented LSMO, the lattice strain is less as compared to (001) oriented LSMO^6^ leading to a smaller distortion of the MnO_6_ octahedra and improved DE interactions. Investigations on interface magnetism have been done before by growing LSMO on (110) and on (001) oriented substrates. They show different orbital occupations of the degenerate e_*g*_ states^7^, mostly guided by the *c*/*a* ratio or the distortion of the MnO_6_ octahedra. For (110) oriented LSMO, the crystal-field variation does not split the ${{\rm{d}}}_{{z}^{2}-{r}^{2}}$ and ${{\rm{d}}}_{{x}^{2}-{y}^{2}}$ orbital states as it does for (001) oriented LSMO. This in turn reduces the antiferromagnetic (AFM) interaction for (110) oriented LSMO but retains for (001) oriented LSMO. This obviously increases the magnetization in (110) oriented LSMO with respect to (001) oriented samples.

#### Interfacial dead layer

The existence of an interfacial dead layer or an antiferromagnetic layer has often been suggested to be responsible for competitions between coexistent phases leading to exotic magnetic properties such as exchange bias^19^. Several mechanisms like, electronic and/or chemical phase separation (structural reconstruction)^20^, Mn orbital ordering (orbital reconstruction) at the interface^3^ and a Mn^3+^ enrichment^21^ (modification of Mn valence state) has been subscribed to the interface layer. We discuss some possible scenarios below.

#### a. Phase change

A LSMO (La_1−*x*_Sr_*x*_MnO_3_) layer with a nominal doping level of x = 0.44 has been found to be ferromagnetic with a moment per Mn atom of 1.5 *μ*_*B*_^22^. A small tensile in-plane strain is sufficient to change the order to an A-type antiferromagnet which can be inferred following the strain versus doping level phase diagrams shown in refs^23,24^. Even though the nominal doping level are designated at x = 0.33, it is often possible to traverse through the phase diagram by oxygen deficiencies and strain and thereby it may be possible to reach the A-AFM region when *c*/*a* < 1^2^. Our samples are grown at low pressure (10^−2^ millibars), which exhibit a reduced magnetization due to oxygen deficiency^20^. Moreover, depending upon the type of strain induced, the antiferromagnet can switch from a C-type to A-type AFM. Strain induced splitting of the e_*g*_ orbital states is common in such structures. It has been shown earlier that ${{\rm{d}}}_{{x}^{2}-{y}^{2}}$ occupancy favors tensile strain (*c*/*a* < 1 or A-type), whereas ${{\rm{d}}}_{{z}^{2}-{r}^{2}}$ occupancy favors compressive strain (*c*/*a* > 1 or C-type). This may give some clues in the understanding of the so-called magnetic “dead” layers at the interfaces^7^. Such a moment reduction has also been attributed earlier to octahedral distortions for thin LSMO layer on STO as octahedral rotations directly affects the structure of the LSMO layer at the interface by increasing its uc symmetry^10^. This effect is similar to the structural phase transition of the bulk material that occurs with substitution of La by Sr.

#### b. Polar discontinuity

As discussed earlier, these behaviors are as expected due to the loss of AFM ordering for the (110) grown samples while presence of AFM ordering for the (001) grown samples^3,7^. The AFM ordering can be due to an interfacial insulating LaSrMnO_3_ layer. Note that below a critical thickness of 10 uc LSMO is in a canted antiferromagnetic insulating phase which coincides with the occurrence of a higher symmetry structural phase with a different oxygen octahedra rotation pattern^10^. This AFM ordering also follows from the fact that (001) oriented LSMO is composed of alternating layers of LaSr-O^+^ and MnO_2_^−^ planes as shown in the sketches in Fig. 7, while BTO is charge neutral with alternating TiO_2_ and BaO layers. The polar discontinuity is attributed to interface electrons that migrate from the LSMO into the conduction band of BTO to solve the “polar catastrophe” of an electric potential that would otherwise diverge with the LSMO thickness. This migration may lead to electronic redistribution affecting the population of the e_*g*_ orbitals and thereby the magnetization^25^. The (110) oriented LSMO, on the other hand, is composed of alternating LaSrMnO^+^ and O_2_^−^ layers and alternating layers of BaTiO^+^ and O_2_^−^ planes. Thus polar discontinuity is avoided and magnetization can be preserved^26^.

#### c. Exchange interaction

The overall scenario can be explained following the reports which indicate that the Mn^3+^ valence state may have a reconstructed e_*g*_ orbital (${{\rm{d}}}_{{z}^{2}-{r}^{2}}/{{\rm{d}}}_{{x}^{2}-{y}^{2}}$) close to the substrate, induced by an in-plane strain (compressive/tensile) of the lattice or by symmetry breaking^3^. We have evidence of mixed valence states in LSMO from the EELS data presented here and x-ray absorption spectroscopy measurements for similarly grown samples. Since we do not observe a lattice strain in BTO in our samples (*c*/*a* ≈ 1), we can probably exclude the effect of strain induced from the BTO underlayer. Thus we are left with symmetry breaking effects which alone can drive the orbital reorganization (favoring the occupation of the e_*g*_(${{\rm{d}}}_{{x}^{2}-{y}^{2}}$) versus the e_*g*_(${{\rm{d}}}_{{z}^{2}-{r}^{2}}$) orbitals when the octahedron is elongated along the x-y plane) of the otherwise energy degenerate Mn 3*d* states. This reorganization can eventually enhance the FM coupling along the *c* axis and AFM interaction along the in-plane axes as the near interface region can segregate into inhomogeneous patches with laterally varying ratio of Mn^3+^/Mn^4+^ ions^27^. This way, the magnetic ordering could vary from AFM (predominantly Mn^3+^ valence) and FM (mixed valence Mn^3+^/Mn^4+^) orders^21^. A superexchange coupling between Mn^3+^–Mn^3+^ sites may lead to a net AFM alignment between the FM regions. This coupling, on the one hand, eventually can reduce the magnetic moment drastically while on the other hand, shows an exchange bias effect along the surface plane. The changes in bond angle and length due to octahedral distortions result in a competition between double exchange and super exchange interactions^10^. The long range spontaneous ordering has been predicted to assist the interface coupling to propagate through the entire thickness of the layer^15^.

## Density functional calculations

To avoid the polar catastrophe at the LSMO/STO interface, the electrons move from inner layers of LSMO to the LSMO side of the interface producing a d^4^ electronic configuration on interface Mn atoms as has been experimentally observed earlier^21,28^. A weakening of the ferromagnetic phase at the LSMO/STO surface was already observed in first principle calculations^29^ for *a* = 3.905 Å, that is larger than the value of bulk LSMO *a* = 3.87 Å. The same effect was obtained using the oxygen vacancies.It was shown that in presence of d^4^ and a > c, an A-type magnetic phase takes place^24^. In our first principle calculation we will demonstrate that, once we have a d^4^ electronic configuration on Mn it is possible to stabilize an A-type phase at the LSMO/BTO interface for large values of *a*. It was already shown that it is possible to reproduce the antiferromagnetic phase in LSMO when you have large strain and a broken symmetry^30^. The AFM coupling is between the Mn planes with shorter distance as in LaMnO_3_.

### First principles calculations

#### Magnetic properties

**Figure 9:**
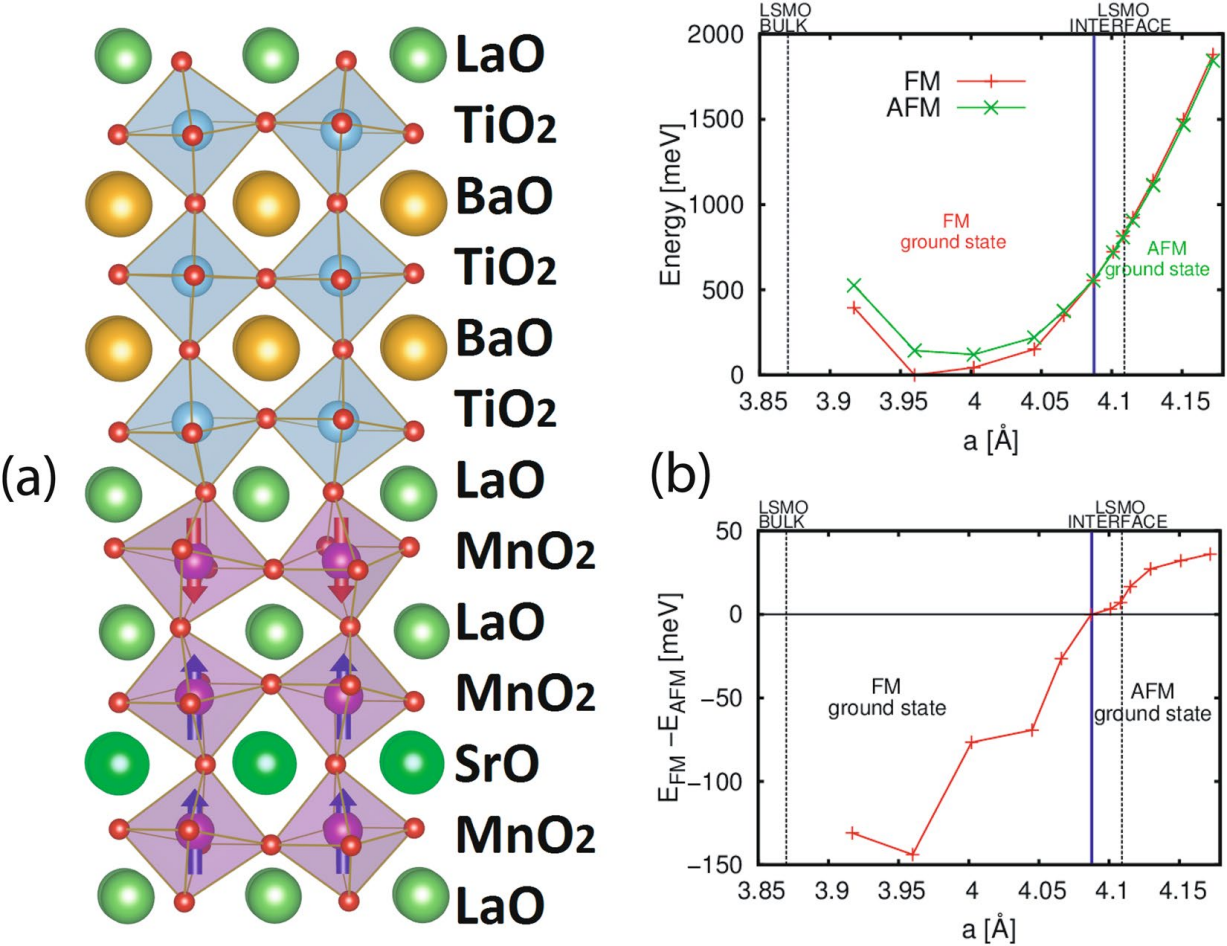
Results of DFT calculations. (**a**) Ground state geometry and magnetic structure of BTO/LSMO heterostructure with 3 BTO layers and 3 LSMO layers. MnO_6_ octahedra are purple while TiO_6_ octahedra are light blue. MnO_6_ octahedra are shown with blue (red) arrows indicating up (down) moments on Mn atoms. Ba, La and Sr atoms are shown respectively as gold, dark green and light green balls. (**b**) Total energy of the ferromagnetic and antiferromagnetic phases of BTO/LSMO as a function of the in plane lattice constant (upper panel). We also report the difference between the two magnetic phases (lower panel). The lattice constant of LSMO and the experimental value of the lattice constant of the system (LSMO interface) are reported in both panels as vertical lines. The vertical solid blue line represents the critical lattice constant value where the magnetic ground state changes.The dashed black vertical lines correspond to the experimental bulk lattice constant of LSMO and to the experimental lattice constant (LSMO INTERFACE) imposed by BTO.

Fig. 9(a) shows the ground state geometry and magnetic structure of BTO/LSMO heterostructure with 3 BTO layers and 3 LSMO layers comprising the supercell structure.The energies of FM and A-type versus in plane lattice constant *a* are shown in Fig. 9(b) (top right panel). The vertical lines correspond to the experimental bulk lattice constant of LSMO and to the experimental lattice constant (LSMO INTERFACE) imposed by BTO. The results indicate a stability region for the A-type phase when a > 4.088 Å, while the experimental in plane lattice constant imposed by the BTO underlayer is *a* = 4.109 Å. We also analyzed other magnetic configurations and we checked that the AFM does not penetrate deeper than a layer into the LSMO side of the interface as shown in Fig. 9﻿(a)﻿ (left panel). The instability towards the A-type magnetic order for particular values of the lattice constant was also found in LSMO/YMO_3_ heterostructure^31^. In Fig. 9(b) (bottom right panel) we plot the energy difference between the FM and AFM phase ver﻿sus in plane lattice constant a.

#### Structural properties

While LSMO presents GdFeO_3_ distortions in the bulk, the BTO presents this kind of distortion at the interface with LSMO. All the Mn-O bonds are inequivalent and there are long (l) and short (s) bonds due to J-T effect. In the bulk AFM phase, the J-T distortion gives (l − s)/(l + s) = 0.069. In Table 1, we show that the J-T effect increased in the AFM phase as happens for the LaMnO_3_ bulk. The La-La interface has large J-T because of the different radius size between La and Sr as we can see in Table 1. Without the large J-T induced by the La we do not have the AFM phase. The antiferromagnetic phase has higher J-T and consequently higher anisotropy. This antiferromagnetic phase with higher anisotropy is present in the sample S3. The magnetic moment increases at the interface as expected for localized electrons.

**Table 1:** Local magnetic moment and $\frac{l-s}{l+s}$ for the three different Mn atoms.

	$\frac{{\boldsymbol{l}}{\boldsymbol{-}}s}{{\boldsymbol{l}}{\boldsymbol{+}}{\boldsymbol{s}}}{\bf{FM}}$	$\frac{{\boldsymbol{l}}{\boldsymbol{-}}s}{{\boldsymbol{l}}{\boldsymbol{+}}{\boldsymbol{s}}}{\bf{AFM}}$	*μ*_*Mn*_ FM	*μ*_*Mn*_ AFM
Inner layer	0.020	0.033	3.55	3.48
La-Sr Interface	0.018	0.029	3.60	3.51
La-La Interface	0.065	0.070	3.60	3.54

#### Electronic properties

**Figure 10:**
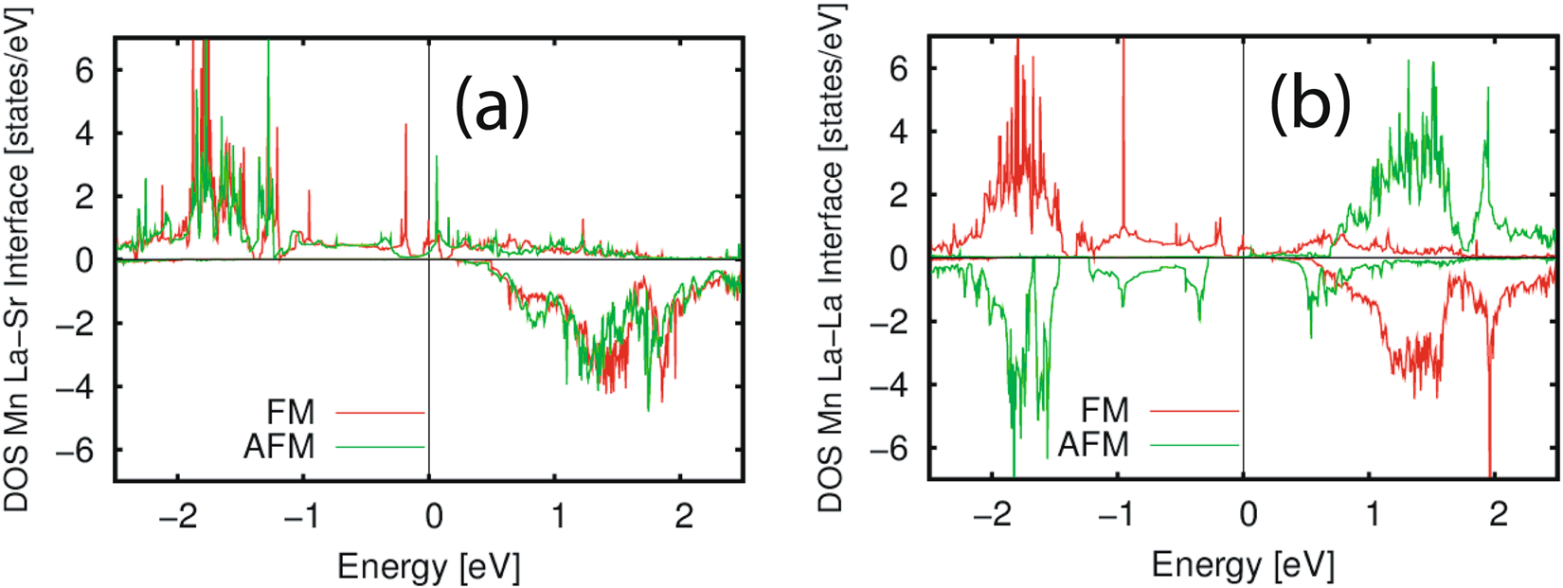
Results of DOS calculations. (**a**) Layer-resolved DOS of the Mn atoms in the superlattice. The upper panel shows the local DOS of Mn atom in the LSMO inner layer, while the lower panel shows the Mn atom at the La-Sr interface. The Fermi energy is set to zero. Spin up (down) contribution are shown in the positive (negative) y-axis. Solid red (green) line represents the DOS of the Mn atoms with FM (AFM) spin configuration. The FM phase shows a half-metallic behavior. The DOS at the Fermi level is strongly suppressed for the AFM phase. (**b**) Layer-resolved DOS of the Mn atoms at the La-La interface in the superlattice. The Fermi energy is set to zero. Spin up (down) contribution are shown in the positive (negative) y-axis. Solid red (green) line represents the DOS of the Mn atoms with FM (AFM) spin configuration. The spin of this atom is up (down) for the FM (AFM) configuration. The FM phase shows a half-metallic behavior. The DOS at the Fermi level is strongly suppressed for the AFM phase.

We calculated the local DOS of the Mn atoms for the La-Sr and La-La interfaces which are shown in Fig. 10(a) (left) and Fig. 10(b) (right). The La-Sr interface is always ferromagnetic. The Mn atoms at the La- La interface present a smaller DOS in the AFM phase though it is not insulating as in the bulk^10^. The metallicity is due to the presence of one ferromagnetic coupling in the heterostructure as shown in Fig. 9(a).

We did not calculate the direction of the spins in our heterostructure, but we assume that this AFM phase has the same bulk properties with spin along the [110] direction^32^. In Fig. 2 of ref.^33^, they show that the exchange bias should be found just in the direction of the spin of the AFM phase. Combining these considerations, the exchange bias should be found predominantly along the [110] direction and should be reduced along the other directions as observed in the experiments.

## Conclusions

Besides epitaxial misfits, which are primarily accommodated by octahedral distortions and/or off-stoichiometry at the LSMO-BTO interface, the layers are often subjected to broken symmetry. Lattice mismatch or symmetry mismatch at the BTO-LSMO interface often leads to interesting functional properties. The aim of this work was to study LSMO-BTO interfaces, especially when they are often subjected to symmetry mismatch rather than lattice mismatch. High quality layers of BTO on (001) and (110) STO substrates were investigated, changing systematically the BTO uc for (001) oriented samples. STEM images indicate in-plane strain for 3 uc thick BTO causing octahedral distortions in LSMO which is due to lattice mismatch. Whereas for thickness >10 uc, the STEM images confirm similar in-plane lattice parameters for coherently grown BTO and LSMO layers in the (001) sample. Here, we were able to identify local lattice distortions of the LSMO uc within a few uc from the interface, which can be attributed solely to the effect of symmetry mismatch.

We found exchange bias when the sample grown on (001) STO is measured along the in-plane [110]/[100] direction with 10 uc of BTO. For samples grown on (110) STO, no such exchange bias exists. PNR measurements confirm the existence of a layer with zero net magnetic moment in the (001) sample showing exchange bias and an overall reduced magnetic moment. The existence of an interfacial antiferromagnetic layer is held responsible for such exotic magnetic properties due to competition between coexistent Mn valence states.

The results of the first-principles DFT calculations indicate that the interface stabilizes an antiferromagnetic phase in the layer closer to the LSMO side. This antiferromagnetic phase is only present at the interface of these two dissimilar oxides and it is not insulating. Our studies suggest that atomic scale tuning of the MnO_6_ octahedra is possible by using symmetry mismatch at heteroepitaxial interfaces.

## Methods

### Sample preparation

Pulsed laser deposition (PLD) was used to grow single-crystalline epitaxial BTO films on TiO_2_ terminated STO (001) substrates and LSMO films on top. A PLD frequency of 5 Hz, energy on targets of around 2 J = cm^2^, spot sizes of 1 × 4 mm^2^ (BTO) and 2 × 4 mm^2^ (LSMO) were used. The substrates were pre-etched to achieve TiO_2_ termination of the STO substrates^34^. The substrate was preheated to 850° C for 30 min and the growth was done at 700° C. The thickness with uc precision was meticulously calibrated using the number of pulses and later on verified using the XRR and TEM data. We have used 125–10000 pulses yielding ~1.25 nm or 3 uc (6 monolayers)–100.0 nm or 250 uc for the BTO layer while 1000 pulses (~15.0 nm) or 39 uc for the LSMO layer in the first batch. A similar pulse counts yield 20 uc (BTO) and 27 uc (LSMO) for the second batch. The pressure during the growth was 1 × 10^−2^ millibars for S1 and S2 and 0.05 × 10^−2^ millibars for S3–S7. Some samples were grown in an oxygen atmosphere (S3, S4, S6, and S7) while others were grown in air (S1, S2, and S5). The oxygen elemental profiles as well as the oxygen concentration, shown in the O-K edge electron energy loos spectrum (EELS) analysis in the supplementary section do not display any significant change from BTO to LSMO for S2 (air-atmosphere) and S3 (oxygen-atmosphere). Local Sr/La ratio change is a major reason to affect Mn valence. As indicated in the EELS data in the supplementary section, there is a local La inhomogeneity as inferred from the Mn valence increase away from the BTO/LSMO interface. Lattice distortion, for example, deformation of the MnO_6_ octahedra, may influence the total energy of Mn^3+^ and Mn^4+^ ions due to J-T effect and hence the probability of their occurrence at the interface. However, small epitaxial misfit may not play significant role on Mn valence as pointed out by previous researchers^35^.

### Sample characterization

#### X-ray characterization

X-ray reflectivity (XRR) and diffraction (XRD) measurements on a Siemens D5000 and a 4-circle D500, respectively, provide information on the layer structure and crystallinity of the layers.

### Transmission electron microcopy

Specimen preparation for transmission electron microcopy (TEM) was carried out using a FEI Quanta 3D dual-beam (SEM/FIB) system at Irvine Materials Research Institute (IMRI), University of California-Irvine (UCI), USA. Typical focus ion beam (FIB) procedures were applied to TEM specimen preparation and low voltage (5 kV) was used for the final thinning to reduce ion-beam-related sample surface damage. TEM samples were examined in a Philips/FEI CM-20 TEM with a LaB6 filament operated at 200 kV and images were recorded using a Gatan CCD camera (Orius 832) and Digital Micrograph software. High-resolution TEM (HRTEM) observations were conducted in a JEOL JEM-2100F TEM equipped with Gatan Oneview camera. TEM/HRTEM experiments enable us to characterize the microstructure of the films and understand the film growth and properties. Scanning TEM (STEM) and electron energy loss spectroscopy (EELS) experiments were conducted at LeRoy Eyring Center for Solid State Science, Arizona State University, using a Probe corrected-JEOL ARM200F S/TEM equipped with Gatan Enfinium EELS spectrometer and NION UltraSTEM 100 with Gatan Enfinium HR spectrometer with high stability electronics.

### Magnetometry

Conventional in-plane magnetization loops were measured at various temperatures and fields using a superconducting quantum interference device (SQUID) from Quantum Design (MPMS-XL).

### Polarized neutron reflectivity

Polarized neutron reflectivity (PNR) measurements for the samples were performed at the reflectometer AMOR in a time of flight (TOF) mode at SINQ, Paul Scherrer Institute in Switzerland. The samples S1 and S2 were measured using the Selene setup at AMOR whereas other samples were measured using the usual setup. In Selene, one uses a convergent beam covering a large angular range instead of a collimated beam by using a 2 × 2 m long elliptically focusing optics in the TOF mode. A resolution of 2 mm was obtained using a position sensitive detector (PSD) positioned about 3 m behind the sample to detect the neutrons. An in-plane magnetic field of 5 kOe was used to saturate the FM layer before the samples were cooled in a closed-cycle cryostat.

From the neutron polarization analysis we resolve the different components of the magnetization within the film plane as only the magnetic moment within the sample plane contributes to the scattering. The scattering length densities (SLD) of a specimen are given by the nuclear (*ρ*_n_) and magnetic (*ρ*_m_) components of the SLD. Two different cross sections were measured namely, the non-spin flip (NSF) channels represented by R_+_ and R_−_. Here + and − signs are used to distinguish the intensity contributions *R* representing a polarization component parallel or anti-parallel to the guiding field, respectively. The NSF scattering amplitude provides information about *ρ*_n_ ± *ρ*_m_ *cosϕ*_A_. We designate *ϕ*_A_ as the angle between the direction of FM magnetization (M_FM_) and the neutron spin quantization axis. The neutron polarization vector is guided by the field applied to the sample (H_a_) along the y-axis. Since we have measured in saturation, *ϕ*_A_ = 0.

### Computational details

We have performed first-principles density functional calculations by using the VASP^36^ package based on plane wave basis set and projector augmented wave method^37^. A plane-wave energy cut-off of 450 eV has been used. For the treatment of exchange-correlation, Perdew-Burke-Ernzerhof^38^ generalized gradient approximation (GGA) has been considered. In order to include strong electron correlations, we have considered a Hubbard U approach^39^, commonly used to describe the electronic structures of correlated oxides. We have considered the U value of 5 eV just for the Ti-*d* atoms^40^ while for Mn-*d* orbitals we have a good descriptions of LSMO and LaMnO_3_-type phase using GGA. The Hund parameter J_*H*_ was kept as 0.75 eV for Ti-*d* atoms. The supercells were built up with 3 layers of BTO and 3 layers of LSMO along the c-axis and a plane $\sqrt{2{\rm{a}}}\times \sqrt{2{\rm{a}}}$ with two Ti atoms as in Fig. 9(a). We calculated the most stable doping configuration of the La_2/3_ Sr_1/3_ O layers. The most stable configuration is with LaO layers and SrO layers in agreement with the literature^31^. The LaO layer prefers to stay at the interface as already shown in other heterostructures of LSMO^31,41^. For simplicity, we have considered a sharp interface in this study.

Many models of interface were tested. We constructed all the possible combinations between the LaO, SrO, BaO layers and the TiO_2_, MnO_2_ layers at the interface searching for an antiferromagnetic phase to compare with the ferromagnetic phase. Besides the ferromagnetic phase, the G-type and A-type antiferromagnetic phases were also studied at the interface. The local formation of BaMnO_3_, LaTiO_3_ or SrMnO_3_ at the interface does not stabilize any AFM phase. To stabilize an AFM phase we study the MnO_2_/LaO/TiO_2_ interface. This choice allows us to have a d^4^ electronic configuration on Mn as observed at the interface^28^. We compare the FM phase with the antiferromagnetic A-type phase shown in Fig. 9(a) where we have an AFM phase at the interface and a FM phase in the inner layers. From the doping point of view, the two interfaces in the supercell are different: in one interface the cage around the Mn atoms is composed by La-La while in the other the cage is composed by La-Sr. We just study the magnetism of the La-La interface because the La-Sr interface is always ferromagnetic. We also analyzed a bigger supercell with 4 BaTiO_3_ and 4 La_2/3_ Sr_1/3_ O layers with MnO_2_/BaO/TiO_2_ getting qualitatively similar results. Bulk phases of LSMO were studied and our results are in agreement with the literature^31^. We find that bulk LSMO is a ferromagnetic metal with a pseudocubic symmetry. However, LSMO is expected to adapt to the BTO underlayer with tetragonal symmetry in the supercell. Though the value of the BTO underlayer was determined experimentally to *a* = 4.109 Å, we study the evolution of the magnetic properties as function of the in plane lattice constant. For every value of the in plane lattice constant a, we calculate the value of c that minimize the total energy separately for both magnetic phases. The geometries were relaxed until the forces on all atoms were reduced to 15 meV/Å. A 6 × 6 × 2 k-points set was used for Brillouin zone integrations in the Monkhorst-Pack scheme for the geometrical relaxation of the heterostructures and a 10 × 10 × 8 k-points mesh for the calculations of density of states (DOS).

## Electronic supplementary material

Atomic-scale engineering of to Supplementary information
