# Publisher Correction: Atomic-scale engineering of ferroelectric-ferromagnetic interfaces of epitaxial perovskite films for functional properties

**DOI:** 10.1038/s41598-017-15018-z

**Published:** 2017-11-10

**Authors:** Simon Hausmann, Jingfan Ye, Toshihiro Aoki, Jian-Guo Zheng, Jochen Stahn, Francis Bern, Binda Chen, Carmine Autieri, Biplab Sanyal, Pablo D. Esquinazi, Peter Böni, Amitesh Paul

**Affiliations:** 1 Technische Universität München, Physik-Department, Lehrstuhl für Neutronenstreuung, James-Franck-Strasse 1, D-85748 Garching, Germany; 2 Division of Superconductivity and Magnetism, University of Leipzig, D-04103 Leipzig, Germany; 3 Laboratory for Neutron Scattering and Imaging, Paul Scherrer Institut, CH-5232 Villigen, Switzerland; 4 Department of Physics and Astronomy, Uppsala University, Box 516, SE-75120 Uppsala, Sweden; 5 Irvine Materials Research Institute, University of California-Irvine, Irvine, CA 92697-2800 USA

*Scientific Reports* **7**:10734; doi:10.1038/s41598-017-10194-4; Article published online 06 September 2017

The original version of this Article contained an incorrect version of the Supplementary Information file. This error has now been corrected in the HTML version of the Article; the PDF version was correct from the time of publication.

