# Supplementary material for: Atomic-scale engineering of ferroelectric-ferromagnetic interfaces of epitaxial perovskite films for functional properties
Source: Sci Rep. 2017 Sep 6;7:10734. doi: 10.1038/s41598-017-10194-4 (PMC5587576; doi:10.1038/s41598-017-10194-4)
Supplement: Supplementary file 1 — Atomic-scale engineering of to Supplementary information [file 41598_2017_10194_MOESM1_ESM.pdf]

Supplementary: Atomic-scale engineering of ferroelectric-ferromagnetic interfaces of epitaxial perovskite films for functional properties

Simon Hausmann[1], Jingfan Ye[1], Toshihiro Aoki[5], Jian-Guo Zheng[5], Jochen Stahn[3], Francis Bern[2], Binda Chen[1], Carmine Autieri[4], Biplab Sanyal[4], Pablo D. Esquinazi[2], Peter Böni[1], and Amitesh Paul[1]*

[1] *Technische Universität München, Physik-Department, Lehrstuhl für Neutronenstreuung, James-Franck-Strasse 1, D-85748 Garching, Germany*

[2] *Division of Superconductivity and Magnetism, University of Leipzig, D-04103 Leipzig, Germany*

[3] *Laboratory for Neutron Scattering and Imaging, Paul Scherrer Institut, CH-5232 Villigen, Switzerland*

[4] *Department of Physics and Astronomy, Uppsala University, Box 516, SE-75120, Uppsala, Sweden and*

[5] *Irvine Materials Research Institute, University of California-Irvine, Irvine, CA 92697-2800, USA*

(Dated: July 20, 2017)

PACS numbers: 75.30.Gw, 75.70.-i

X-ray reflectivity (XRR) and X-ray diffraction (XRD)

Fig. 1(a,b,c) shows the XRR data (scan along Q_z) for the samples S1, S3–S7 grown on (001) and S2 grown on (110) STO. The reflectivity spectra were analysed by means of a standard fitting routine based on the Parratt algorithm[1]. The film was modelled as consisting of individual layers of specific thickness, roughness and scattering length density. The simulations reveal that the thicknesses of the layers are close to their nominal values. We find an additional layer (2.0 nm) on top of the LSMO layer with a reduced SLD when compared to the rest of the LSMO layer. This can be due to Sr segregated phases of LSMO[2].

Room temperature XRD measurements at Cu-Kα wavelength have been performed to probe the crystallinity of the films. Fig. 1(d,e) shows the representative XRD data for the samples (a) S1, S2 and (b) S3, S5, S7. All samples show thicknesses close to nominal thickness and high crystalline quality with (110) and (00l) orientations of the LSMO and BTO films[3]. The Laue oscillations corresponding to the BTO and LSMO peaks are visible at least for S3 and S5 indicating the bilayer coherency accounting for the number of contributing layers. The reflections are associated with a background created by Bremsstrahlung. The out-of-plane lattice constant for the (001) grown sample S1 is around c = 4.045 Å (BTO). The out-of-plane lattice constants for the (110) grown sample S2 are d = 2.71 Å (LSMO) and d = 2.841 Å (BTO) as obtained from the XRD data. The out-of-plane lattice constant of BTO in S7 is c = 4.072 Å and the in-plane lattice constant is a = 4.09 Å, indicating that the BTO film grown on STO has a pseudocubic structure or an unstrained TiO_6 octahedron with c/a ratio = 0.996≈1, as compared to bulk BTO where c/a = 1.01. The out-of-plane lattice constant of the LSMO film in S3 is around c = 3.861 Å and the in-plane constant a = 4.033 Å, confirming that the LSMO film on BTO is under an in-plane tensile strain as c/a = 0.9527. A systematic variation of the lattice parameters and thereby strain in LSMO with BTO thickness could not be ascertained. The out-of-plane lattice parameter c was estimated from the 002 reflection while the in-plane lattice parameter a (at a tilt angle χ=55.25°) was estimated from the 111 reflection. A schematic of the respective lattice parameters from the XRD data are also shown in Fig. 1(f).

Lattice mismatch and symmetry mismatch from Z-contrast TEM images

The in-plane lattice parameters were extracted from the Z-contrast images of cross-sectional TEM specimens of the coherently grown samples S3 and S5 and are shown in Fig. 2(a,c) as a function of the number of monolayers. The measurements were done for several unit cells (uc) and the average of the lattice parameters a were calibrated using $a =$ 3.905 Å in STO within two different regions A and B for each sample. The average a-axis parameters show little variation from 3.909 Å in BTO to 3.906 Åin LSMO for S3. In S5, it varies from 3.932 Å in BTO to 3.898 Å in LSMO. One may note that in S3, the change in a parameter is accommodated within 3 uc of the LSMO layer at the interface while in S5, it is accommodated well within the BTO layer itself.

As the uc of the film attempts to accommodate the in-plane lattice parameter of the underlying single crystal substrate, the ABO_3 perovskite structural unit deforms causing distortions of the BO_6 octahedra. Strain is accommodated by changing the layer's uc size via rotations of corner-connected MnO_6 units. It has been shown earlier that lack of rotations at the LSMO/STO (001) interface and subsequent unusual c-axis lattice expansion (by 0.025 Å) is not caused by conventional lattice mismatch but is rather induced by crystallographic symmetry mismatch between LSMO and STO structures across the interface[4]. In ultrathin LSMO films grown on (110)-oriented STO substrates, however, the octahedral coupling across the interface induces distinctive BO_6 octahedral distortions leading to lattice mismatch rather than symmetry mismatch. In our samples S3 and S5, we have measured the out-of-plane lattice parameters along the c-axis as a function of layer uc from the Z-contrast images of cross-sectional TEM specimens as shown in Fig. 2(b,d).

For S3, the average c-axis parameter within the BTO layer changes from 4.013 Å to 3.94 Å within LSMO which further reduces to 3.9 Å beyond 3 uc. Similarly, the c/a ratio varies within the BTO layer from 1.03 at the STO-BTO interface to 1.0 within the LSMO layer near the BTO-LSMO interface before it stabilizes at 0.99. This is a typical consequence of lattice mismatch.

For S5, the average c-axis parameter within the BTO layer changes from 4.229 Å for the initial 3 uc to 3.892 Å within 2-3 uc and is extended over the next 5 uc. The c-axis parameter in BTO decreases gradually with distance from STO/BTO interface, which is a substrate effect. It shows a jump to 3.973 Å within the LSMO layer and is extended over for at least

6 uc away from the interface. The c/a ratio varies within the BTO layer from 1.08 at the STO-BTO interface to close to 1.0 near the BTO-LSMO interface. It increases again to 1.01 within the LSMO layer but reduces to 1.0 farther away from the interface where it stabilizes. This LSMO c-axis parameter expansion (by 0.081 Å) at the interface cannot be explained by any octahedral tilts and rotations but can be a consequence of symmetry mismatch at the BTO-LSMO interface of S5. In order to maintain corner connectivity between TiO_6 and MnO_6 octahedral units across the interface, the LSMO uc is forced to adapt the symmetry of the underlying BTO layer. Farther from the interface, the film structure acquires octahedral tilts, leading to a reduced c-axis parameter.

Scanning tunneling electron microscopy (STEM) and Electron energy loss spectroscopy (EELS)

EELS together with Z-contrast STEM was further used to characterize Mn valence in sample S2, as an example. In LSMO, Mn valency changes with local Sr (La) concentration, which is closely related to the physical property of LSMO[5]. The Mn valence can be studied by using either Mn L-edge or oxygen K-edge fine structures with different methods. Here we try to determine the Mn valence using the energy difference (ΔE) of the 1st and 2nd peaks in oxygen K-edge fine structure because ΔE is not sensitive to sample thickness and is easy to measure[6]. Fig. 3(a) shows a Z-contrast STEM image of the sample area, from which the dots labeled with numbers indicate the locations where the EELS spectra were acquired. Figure 3(b) displays the energy-loss near-edge structure (ELNES) of oxygen K-edges labeled with location number at the left side of each spectrum. These spectra are extracted from an EELS line scan along the dotted line (Fig. 3(a)) in the film growth direction, starting from the STO substrate and ending at the top of LSMO film. The spot size used in line scan was 0.1 nm and an average distance between each spectrum was about 0.085 nm to ensure over sample. In order to improve the signal to noise ratio, five spectra (two spectra before a dot and two spectra after a dot) were summed into one spectrum, and plotted in Fig. 3(b), averaging a few spectra acquired nearby the labeled dots. To make sure that the electron beam condition and sample thickness are suitable for EELS analysis, some spectra from STO substrate and BTO layer are included. Spectra 1 and 3 display typical oxygen K-edges of STO and BTO, respectively. Spectra 4 and 4.5 are from the overlapping area

of BTO and LSMO, while spectra 5–8 are obtained from the LSMO layer. The positions to obtain the Mn-related spectra are also marked in the elemental profiles of the sample (Fig. 3(c)). The spectrum 4 shows features similar to the spectrum 3, while the spectrum 4.5 resembles spectrum 5. There are three prominent peaks labeled as A (1st peak), B (2nd peak) and C (3rd peak) in each spectrum from the LSMO film. Peaks A and C are due to hybridization of the oxygen $2p$ states with the transition metal (*i.e.* Ti and Mn) $3d$ and $4sp$ states, respectively, while peak B reveals characteristic of oxygen $2p$ states hybridized with Sr $4d$ or La $5d$ states[7,8]. What we concern is the energy difference (ΔE) between peaks A and B, which can be used to determine Mn valence[9]. In order to see the ΔE clearly, the spectra are displaced vertically and the energy scale has been shifted so that the A-peaks are aligned. The measured ΔE value is also marked close to the B-peak in each spectrum. The LSMO film can be approximately divided into three regions according to the ΔE value. The 1st LSMO region with ΔE=4.8 eV is overlapped with BTO and has a thickness of about 3 nm. The 2nd LSMO region with ΔE=5.2 eV–5.3 eV has a thickness of about 5 nm and the 3rd region with ΔE=5.7 eV–5.9 eV is on top of the 2nd region. The Mn valence can be determined approximately by the ΔE value[8]. So the 3rd LSMO region has the Mn valence close to the normal value 3.33^{+} ($La_{0.67}Sr_{0.33}MnO_3$ with x=0.33) or slightly higher. The 2nd LSMO region has a smaller Mn valence which is estimated to be about 3.27. The 1st LSMO region has Mn valence of about 3.20. Mn L_3/L_2 white line intensity ratio ($L_{2,3}$ ratio) is also used to examine Mn valence. Fig. 3(d) shows the Mn $L_{2,3}$ ratio at locations from spot 4 to spot 8, which decreases when the distance between the BTO/LSMO interface and detection location increases. The ratio is calculated using the integrated signal under L_3 and L_2 lines, which was corrected by removing the background below the Mn $L_{2,3}$ ELNES using a power law and the continuum contribution using a Hartree-slater cross-section step function. The detailed procedure is similar to the one done by Varela *et al.*[6]. We do not intend to quantify the Mn valence using the $L_{2,3}$ ratio because the ratio is often affected by sample thickness and other factors, but the trend of $L_{2,3}$ ratio supports the conclusion drawn from O-K ELNES analysis, that is, the Mn valence increases as the $L_{2,3}$ ratio decreases with detection distance from the BTO/LSMO interface[6].

Mn valence is influenced by several factors such as local composition, strain, magnetic structure and phase segregation. We carried out FFT of lattice images in all observed LSMO areas. No phase segregation was observed. The O concentration (Fig. 3(c)) does not display

any significant change from BTO to LSMO. Local Sr/La ratio change is a major reason to affect Mn valence. As indicated in the main section, there is a local La inhomogeneity. The EELS line scan (Fig. 3(c)) shows a good correlation between the La signal (in yellow) and Mn valence. The 2nd LSMO region with a lower Mn valence where spots 5 and 6 are located has a slightly higher La concentration than the 3rd LSMO region with the normal Mn valence where spots 7 and 8 are located. This implies that the Sr/La ratio could be lower in the 2nd layer than that in the 3rd layer although Sr concentration is not known because of very weak Sr EELS signal. Lattice distortion, for example, deformation of the MnO_6 octahedra, may influence the total energy of Mn^{3+} and Mn^{4+} ions due to J-T effect and hence the probability of their occurrence at the interface. However, small epitaxial misfit may not play significant role on Mn valence as pointed out by previous researchers[8].

In order to show the effect of interdiffusion on the BTO thickness we show the EELS elemental line profiles of S3 and S2 in Fig. 4(a,b). One can see that with increase in uc number 3 in S3 and 20 in S2, the interdiffusion has increased from less than 1 nm to around 5 nm.

Furthermore, the Mn L-edge EELS spectra was used to characterize the Mn valence along the growth direction for S5 (BTO 10) with (001) orientation. Fig. 5(a,b) shows a plot of Z-contrast STEM image of cross-sectional sample S5 and the corresponding Mn L-edge profiles as a function of monolayers across the LSMO layers at the heterointerfaces. The spectra exhibit no significant shift in the energy or shape of the Mn L-edge (from layer numbers 4 to 7) which indicates a homogeneous mixed valence state of Mn across the bilayer without charge transfer. This confirms that the magnetic properties described here arise from structural modulations and are not from charge-based effects.

Magnetization

The magnetization measurements were done along two crystallographic axes: at 0° [100] along the y-axis and at 45° along [110] with respect to the y-axis, rotating the magnetic field direction in the film plane. An in-plane rotation showed an easy axis along the [110] direction[10]. The growth direction is designated along the z-axis.

Here, we focus on the second batch of samples. The 001 samples, S3–S7, were field cooled (FC) at various fields and zero field cooled (ZFC) and measured on heating at various fields as

a function of temperature. FC at 100 Oe and ZFC magnetization versus temperature curves are shown in Fig. 6(a–e). One can observe a peak in the ZFC curves (T_p) and a bifurcation between the ZFC and FC curves below the irreversibility temperature T_F. These can be characteristics of superparamagnetic (SPM) or super spin glass (SSG) or superferromagnetic (SFM) type of behavior[11]. When the inter-particle interaction dominates over single particle blocking it may lead to a collective freezing behavior like SSG or SFM. The net magnetic moment is also seen to be relatively reduced in S5 and S6 as compared to the other samples.

The two characteristic temperatures, T_p and T_F, are very close, indicating a blocked or frozen state. The broad peak around T_p, signifies a broad distribution of the blocking temperature. Fig. 6(f) shows the variation of T_p and T_F with BTO thickness. We notice a distinct decrease in T_p with decreasing BTO thickness which indicates the influence of strain on the LSMO magnetization and the variation of the average blocking temperature of the clusters. Earlier, signatures of a superparamagnetic–type of behavior was noticed for similar samples[3].

In Fig. 6(g–k) we show the hysteresis loops measured along the [110] axis of samples S3–S7 at T = 10 K, with a cooling field $H_{\mathrm{FC}} = 10$ kOe. We plot H_{c} and H_{EB} as a function of BTO thickness in Fig. 6(l).

---

* Corresponding author; Electronic address: `amitesh.paul@frm2.tum.de`

1 L. G. Paratt. Surface Studies of Solids by Total Reflection of X-Rays. Phys. Rev. **95**, 359 (1954).

2 R. Herzer *et al.*, Structure determination of monolayer-by-monolayer grown $La_{1-x}Sr_xMnO_3$ thin films and the onset of magnetoresistance. Phys. Rev. B **77**, 085401 (2008).

3 R. Flaschmann *et al.*, Self-organized in-plane ordering of nanostructures at epitaxial ferroelectricferromagnetic interfaces. J. Appl. Cryst. **49**, 1693 (2016).

4 A. Vailionis *et al.*, Symmetry and lattice mismatch induced strain accommodation near and away from correlated perovskite interfaces. Appl. Phys. Lett. **105**, 131906 (2014).

5 A. Urushibara *et al.*, Insulator-metal transition and giant magnetoresistance in $La_{1-x}Sr_xMnO_3$. Phys. Rev. B **51**, 14103 (1995).

6 M. Varela *et al.*, Atomic-resolution imaging of oxidation states in manganites. Phys. Rev. B **79**, 085117 (2009).

[7] F. Pailloux *et al.*, Nanoscale analysis of a $SrTiO_3/La_{2/3}Sr_{1/3}MnO_3$ interface. Phys. Rev. B **66**, 014417 (2002).

[8] T. Riedl, T. Gemming, K. Dörr, M. Luysberg, K. Wetzig. Mn Valency at $La_{0.7}Sr_0.3MnO_3/SrTiO_3$ (001) thin film interfaces. Microsc. Microanal. **15** , 213 (2009).

[9] M. Abbate *et al.*, Controlled-valence properties of $La_{1-x}Sr_xFeO_3$ and $La_{1-x}Sr_xMnO_3$ studied by soft-x-ray absorption spectroscopy. Phys. Rev. B **46**, 4511 (1992).

[10] A. Alberca *et al.*, Magnetoelastic coupling in $La_{0.7}Ca_{0.3}MnO_3/BaTiO_3$ ultrathin films. Phys. Rev. B **88**, 134410 (2013).

[11] S. Mukherjee *et al.*, Exchange-bias-like coupling in a Cu-diluted-Fe/Tb multilayer. Phys. Rev. B, **91**, 104419 (2015).

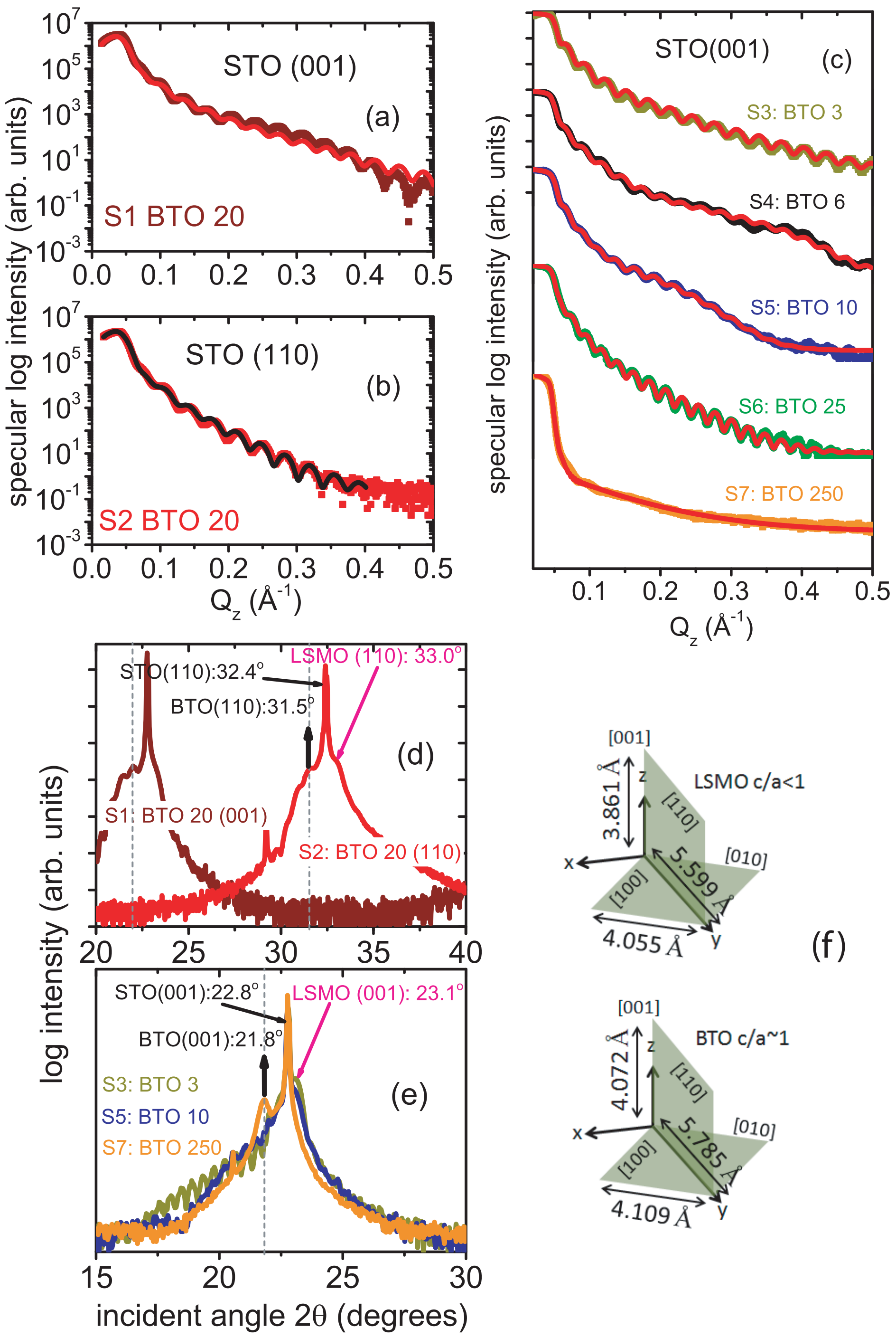

FIG. 1: (Color online) **XRR and XRD patterns of the specimens. (a,b,c)** XRR (Cu-K_α) patterns of the samples S1, S3–S7 grown on (001) and S2 grown on (110) STO and their fits are plotted versus Q_z at room temperature. (**d**) XRD patterns of S1 and S2 of the first batch and (**e**) S3, S5 and S7 of the second batch with increasing BTO uc **(f)** A sketch of the respective lattice parameters as obtained from the XRD data of S3 (LSMO) and S7 (BTO) are also shown alongside.

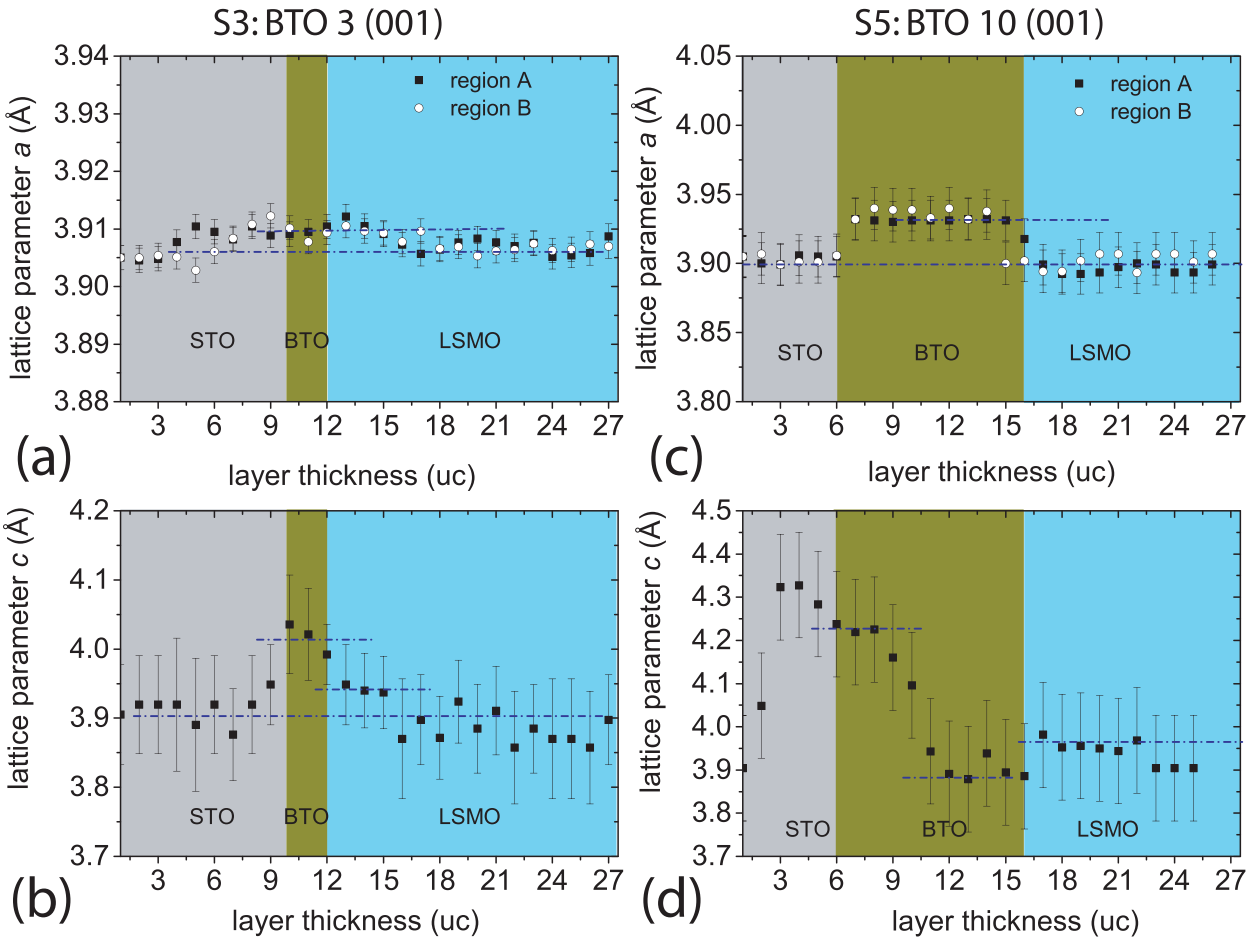

FIG. 2: (Color online) **Lattice parameters of the specimens.** The plot of the extracted lattice parameters as a function of the number of uc from the Z-contrast images of cross-sectional TEM specimens. Each data point was obtained by averaging a set of 10 measurements from unit cells arranged parallel to the interface. The error bars indicate the standard error of the measurements. (a) In-plane lattice parameters along the a-axis for S3, (b) out-of-plane lattice parameters along the c-axis for S3, (c) in-plane lattice parameters along the a-axis for S5 and (d) out-of-plane lattice parameters along the c-axis for S5. The blue lines are a guide to the eye.

S2: BTO 20 (110)

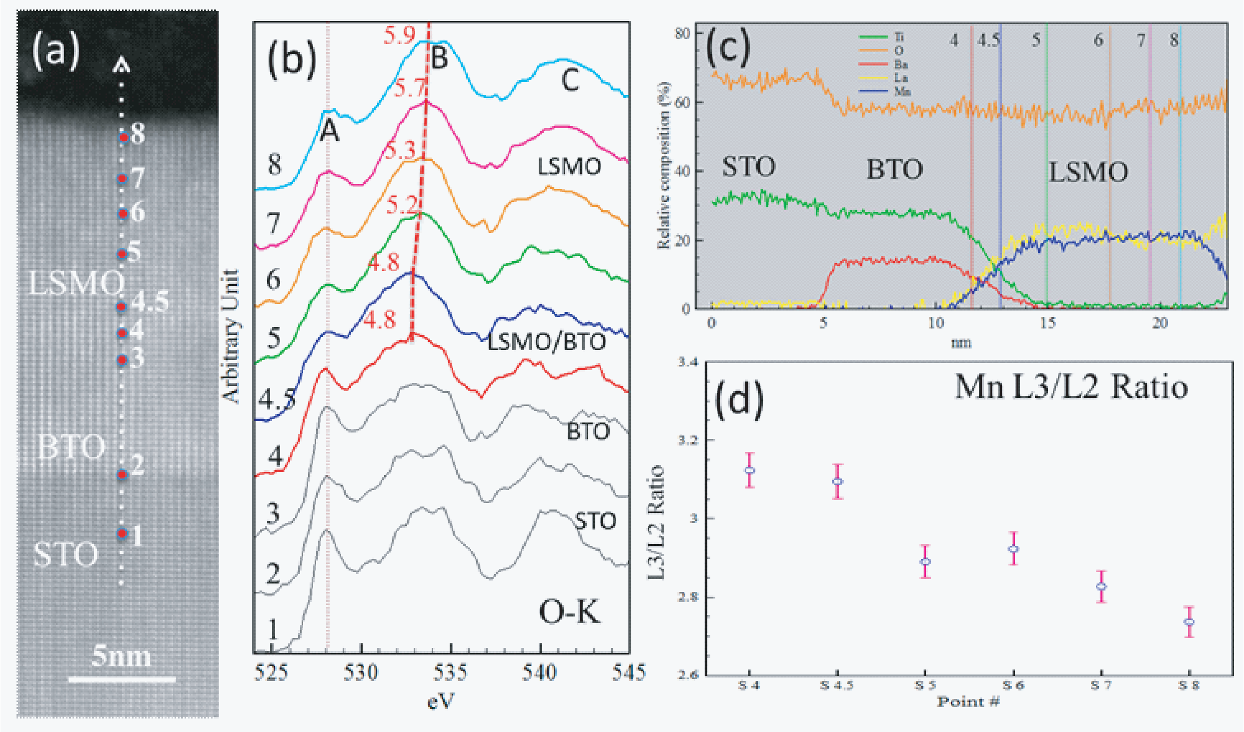

FIG. 3: (Color online) **EELS data of specimen S2.** (a) Z-contrast STEM image of cross-sectional sample S2. The dots labeled with numbers indicate the locations where the EELS spectra were acquired. (b) ELNES of oxygen K-edges labeled with location number at the left side of each spectrum. (c) Elemental profiles along the dotted line in (a). (d) Mn L_3/L_2 white line intensity ratio across the LSMO layer which is used to examine Mn valence.

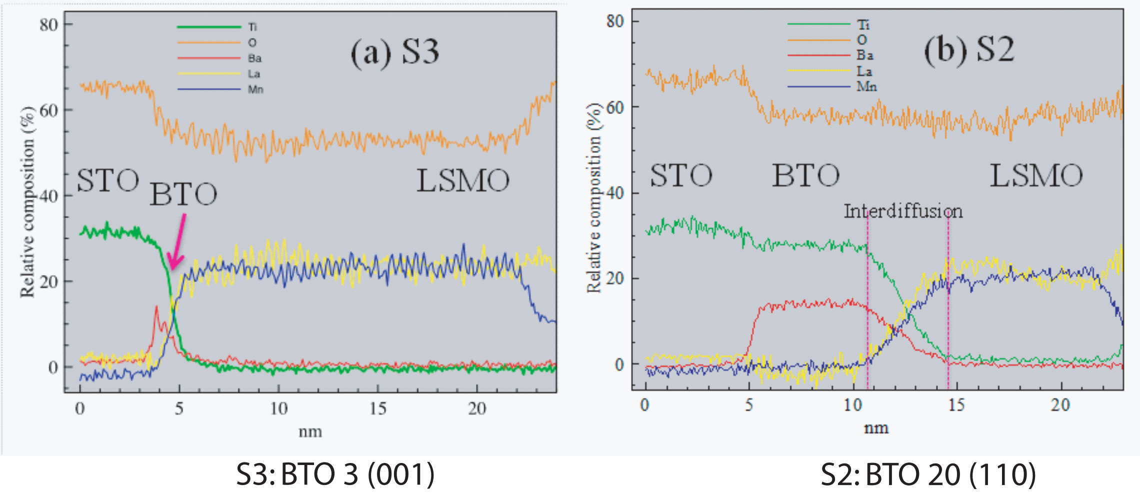

FIG. 4: (Color online) **EELS elemental profiles of the specimens.** The plot of EELS elemental profiles for two different thicknesses of BTO corresponding to the samples (a) S3 and (b) S2.

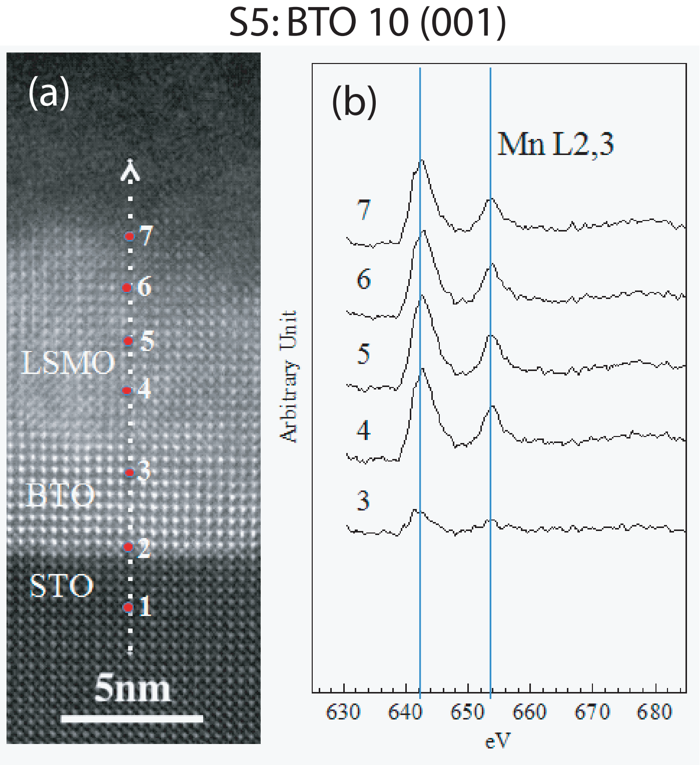

FIG. 5: (Color online) **EELS profile for S5.** (a) Z-contrast STEM image of cross-sectional sample S5 (BTO 10) and (b) the corresponding EELS Mn L-edge profiles across the layers of LSMO.

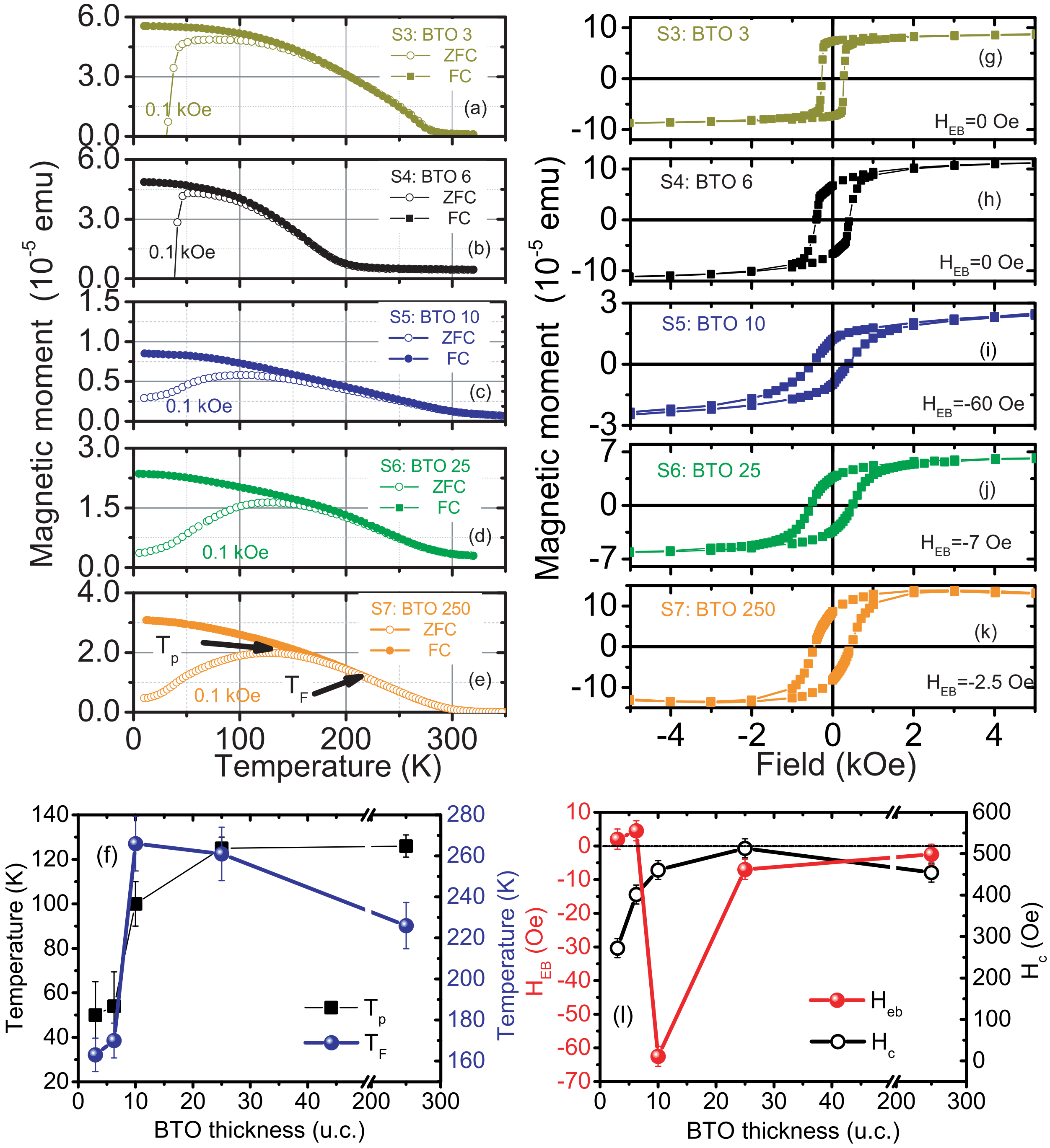

FIG. 6: (Color online) **Magnetization measurements of the specimens.** The plot of FC and ZFC measurements for the (a) S3 (b) S4 (c) S5 (d) S6 and (e) S7 samples measured on heating at 100 Oe. (f) Plot of T_p and T_F versus BTO thickness. Hysteresis loop measurements at 10 K for the (g) S3 (h) S4 (i) S5 (j) S6 and (k) S7 samples measured along the [110] direction after field cooling in 10 kOe along the same direction. (l) Plot of H_c and H_{EB} versus BTO thickness.
